# Effectiveness of Telemedicine for Musculoskeletal Disorders: Umbrella Review

**DOI:** 10.2196/50090

**Published:** 2024-02-02

**Authors:** Silvia Bargeri, Greta Castellini, Jacopo Antonino Vitale, Stefania Guida, Giuseppe Banfi, Silvia Gianola, Federico Pennestrì

**Affiliations:** 1 Unità di Epidemiologia Clinica IRCCS Istituto Ortopedico Galeazzi Milan Italy; 2 Spine Center Schulthess Klinik Zürich Switzerland; 3 IRCCS Istituto Ortopedico Galeazzi Milan Italy; 4 Università Vita-Salute San Raffaele Milan Italy

**Keywords:** telemedicine, systematic review, patient-reported outcomes measures, PROMs, patient-reported experience measures, PREMs, rehabilitation, musculoskeletal disorder, condition, musculoskeletal, patient-reported, telerehabilitation, orthopedics, meta-analyses, osteoarthritis, accessibility, health care

## Abstract

**Background:**

Several systematic reviews (SRs) assessing the use of telemedicine for musculoskeletal conditions have been published in recent years. However, the landscape of evidence on multiple clinical outcomes remains unclear.

**Objective:**

We aimed to summarize the available evidence from SRs on telemedicine for musculoskeletal disorders.

**Methods:**

We conducted an umbrella review of SRs with and without meta-analysis by searching PubMed and EMBASE up to July 25, 2022, for SRs of randomized controlled trials assessing telemedicine. We collected any kind of patient-reported outcome measures (PROMs), patient-reported experience measures (PREMs), and objective measures, including direct and indirect costs. We assessed the methodological quality with the AMSTAR 2 tool (A Measurement Tool to Assess systematic Reviews 2). Findings were reported qualitatively.

**Results:**

Overall, 35 SRs published between 2015 and 2022 were included. Most reviews (n=24, 69%) were rated as critically low quality by AMSTAR 2. The majority of reviews assessed “telerehabilitation” (n=29) in patients with osteoarthritis (n=13) using PROMs (n=142 outcomes mapped with n=60 meta-analyses). A substantive body of evidence from meta-analyses found telemedicine to be beneficial or equal in terms of PROMs compared to conventional care (n=57 meta-analyses). Meta-analyses showed no differences between groups in PREMs (n=4), while objectives measures (ie, “physical function”) were mainly in favor of telemedicine or showed no difference (9/13). All SRs showed notably lower costs for telemedicine compared to in-person visits.

**Conclusions:**

Telemedicine can provide more accessible health care with noninferior results for various clinical outcomes in comparison with conventional care. The assessment of telemedicine is largely represented by PROMs, with some gaps for PREMs, objective measures, and costs.

**Trial Registration:**

PROSPERO CRD42022347366; https://osf.io/pxedm/

## Introduction

Telemedicine is a broad term encompassing many applications, such as diagnostic asynchronous evaluation, continuous monitoring using biosensors, and synchronous video consultations, including multiple variations on each theme. This definition includes “telerehabilitation,” “health technologies,” “digital medicine,” and similar keywords [1,2]. During the COVID-19 pandemic, the spread of smart devices and accessible internet connections made telemedicine grow exponentially, become increasingly popular, and be tested for many health conditions [3,4]. Musculoskeletal disorders usually require multidisciplinary and multifacility treatment throughout different settings and providers (physiotherapy, rehabilitation, prehabilitation, and orthopedics; inpatient, outpatient, and home); therefore, the adoption of telemedicine can improve clinical and patient-reported outcomes along with organizational arrangements and cost savings [5,6]. Telemedicine in rehabilitation first appeared in a scientific publication in 1998 [7]; rehabilitation is an old branch of medicine, and in the last 20 years, new telemedicine practices have been developed showing an interest in understanding its effectiveness. However, there is not yet a universal definition of telemedicine nor a consensus on its effects.

In addition, in recent years, an increasing number of studies have used patient-reported outcome measures (PROMs) and patient-reported experience measures (PREMs) to evaluate telemedicine services [8]. With the increasing maturity of telemedicine applications and higher evidence levels, the use of PROMs has increased. PREMs in turn are useful to describe the health care service experience from the perspective of patients [9-11] in order to identify real-world factors (eg, organizational, relational, environmental) that may improve or hamper the access to, quality of, and safety of care [12]: in the case of telemedicine, the user-friendliness of a certain technology, its actual functioning in ordinary settings (eg, considering backlogs and poor internet connections), the clarity of the instructions received, and the degree of interoperability among different providers (health care facilities, professionals, and technology suppliers). Otherwise, excellent technical care may be wasted by lack of compliance, poor health literacy, and insufficient patient engagement.

Different systematic reviews (SRs) [13-15] have been published examining different types of telemedicine solutions in specific musculoskeletal populations. However, it is crucial to investigate evidence on not just a single question but across multiple questions pertaining to a specific topic [16]. This emphasizes the importance of providing the best available evidence on the effectiveness of telemedicine and telerehabilitation in the entire musculoskeletal field, encompassing all telemedicine applications.

The aim of this umbrella review is to explore the effectiveness of telemedicine and rehabilitation in the treatment of musculoskeletal conditions in terms of physical impairment, function, health-related quality of life (HRQoL), adverse events, adherence, and costs, including PROMs and PREMs. This would help professionals improve decision-making and yield better clinical outcomes for patients.

## Methods

### Study Design

We conducted an umbrella review according to the Cochrane Handbook’s chapter on overviews of reviews and the *JBI Manual for Evidence Synthesis*. Reviews of SRs are referred to by several different names in the scientific literature, including “umbrella reviews,” “overviews of reviews,” “reviews of reviews,” “summaries of systematic reviews,” and “synthesis of reviews” [17,18]. We followed the Preferred Reporting Items for Systematic Reviews and Meta-Analyses (PRISMA) [19] guidelines for the flow chart and the Preferred Reporting Items for Overviews of Reviews (PRIOR) [20,21] as a reporting checklist. The review protocol was registered in the International Prospective Register of Systematic Reviews (PROSPERO) database (CRD42022347366).

### Criteria for Considering Reviews for Inclusion

According to Cochrane’s definition, an SR is a review of the literature that “attempts to identify, appraise and synthesize all the empirical evidence that meets pre-specified eligibility criteria to answer a specific research question by using explicit, systematic methods that are selected with a view aimed at minimizing bias, producing more reliable findings to inform decision making” [22]. In this umbrella review, we considered inclusion criteria according to the PICOS (population, intervention, comparison, outcomes, study design) format: patients with any musculoskeletal or orthopedic condition (population); any kind of interventions based on advanced technology systems named as “telemedicine,” “telerehabilitation,” “health technologies” and “digital medicine,” delivered both in synchronous and asynchronous modalities (intervention); in-person treatment or usual care or no treatment (comparison); PROMs, PREMs, or objective measures (outcomes); SRs of randomized controlled trials (RCTs) (study design).

SRs were excluded if they assessed (1) observational studies, (2) mixed populations (eg, if they combined effects for cardiovascular and musculoskeletal patients, or if it was not possible to separate data for a population of interest, such as musculoskeletal patients), or (3) interventions that aimed to configure technical aspects of devices and apps.

### Main Outcomes

We considered outcomes related to the following domains: physical impairment, function, health-related quality of life (HRQoL), adverse events, and adherence to therapy or care pathways, expressed as the following categories: PROMs, PREMs, or objective measurements. PROMs are used to assess a patient’s health status at a particular point in time (eg, common symptoms, pain, stiffness, HRQoL, and disease-specific interference with domestic activities and leisure time). Furthermore, we considered PREMs as any patient-reported information about the experience of treatment (eg, inclusivity of a technology, adequate communication with health care professionals, availability of professionals, possibility to ask questions, clarity of information received, spared visits to hospital, access to treatment, and perceived safety). Costs related to treatments were also collected.

### Search Strategy

A search of SRs was performed in PubMed and Embase from inception to July 25, 2022. The search was restricted to English-language publications. No restriction on year was applied (Multimedia Appendix 1 [8,23], Table S1).

### Data Extraction (Selection and Coding)

Two independent reviewers consulted information screening sources by title and abstract. The full text of relevant studies was downloaded and evaluated for final inclusion according to the inclusion criteria. Any conflict was resolved through discussion. EndNote (version 20; Clarivate) and Rayyan (Qatar Computing Research Institute) were used to manage the study selection phase. The selection process is shown in the PRISMA flow chart [24].

Two independent reviewers extracted general characteristics of the SRs (eg, country, funding, and conflicts of interest), population characteristics, interventions, comparisons, and outcomes. Patient populations were classified according to the International Classification of Diseases, 10th revision (ICD-10) codes for diseases of the musculoskeletal system and connective tissue (M00-M99) [25].

Interventions were classified into three categories, building on a published taxonomy [8]: (1) teleconsultation (providing health care over a distance), telediagnostics (identifying diseases over a distance), and telemonitoring (collecting data over a distance to allow medical decisions); (2) telerehabilitation (collecting data over a distance to help patients cope with the long-term consequences of disease or impairment); and (3) digital self-management (to promote patient health responsibility and encourage health literacy).

Outcomes were also classified into subgroups for 3 categories: PROMs, PREMs, and objective outcomes. Among PROMs, we examined the following outcome domains: pain, HRQoL, physical function, social function, emotional function, cognitive function, health literacy, side effects, and adherence. We categorized PREMs into 2 outcome domains: treatment and technology. In Multimedia Appendix 1 [8,23], Table S2, we report all details about PROM and PREM taxonomy [8].

Objective measures were mapped into the same categories (eg, physical function) and costs.

Quantitative results were extracted from meta-analyses if reviews included more than 1 study in each analysis. For continuous outcomes, we extracted mean difference (MD) or standardized mean difference (SMD) with their 95% CIs. For binary outcomes, we extracted odds ratios (ORs) or relative risks (RRs) with their 95% CIs. In absence of quantitative syntheses, results were reported descriptively.

### Risk of Bias (Quality) Assessment

The methodological quality of reviews was assessed using AMSTAR 2 (A Measurement Tool to Assess systematic Reviews 2) [26]. It consists of 16 items rating the quality of each SR as high, moderate, low, or critically low. Two reviewers independently performed the assessments; a third reviewer resolved any disagreement between reviewers.

### Strategy for Data Synthesis

We followed the methodology outlined in the *Cochrane Handbook* chapter on overviews of reviews and the *JBI Manual for Evidence Synthesis* [18]. The characteristics of the included SRs were described with narrative synthesis. The main results reported in the reviews at the shortest follow-up were summarized. Qualitative results reported by the reviews were narratively summarized, while meta-analyzed results were visually presented in terms of directions of effects (favor intervention, no difference between groups, favor control) by a bubble plot map in which bubbles were organized into subgroups based on the AMSTAR 2 assessment, proportional to the number of participants for each meta-analysis and colored by outcomes and population [27].

Since we expected heterogeneity across reviews due to the different populations, interventions, controls, and outcomes included, the number of overlapping primary studies included in the SRs was not assessed.

## Results

### Study Selection

**Figure 1:**
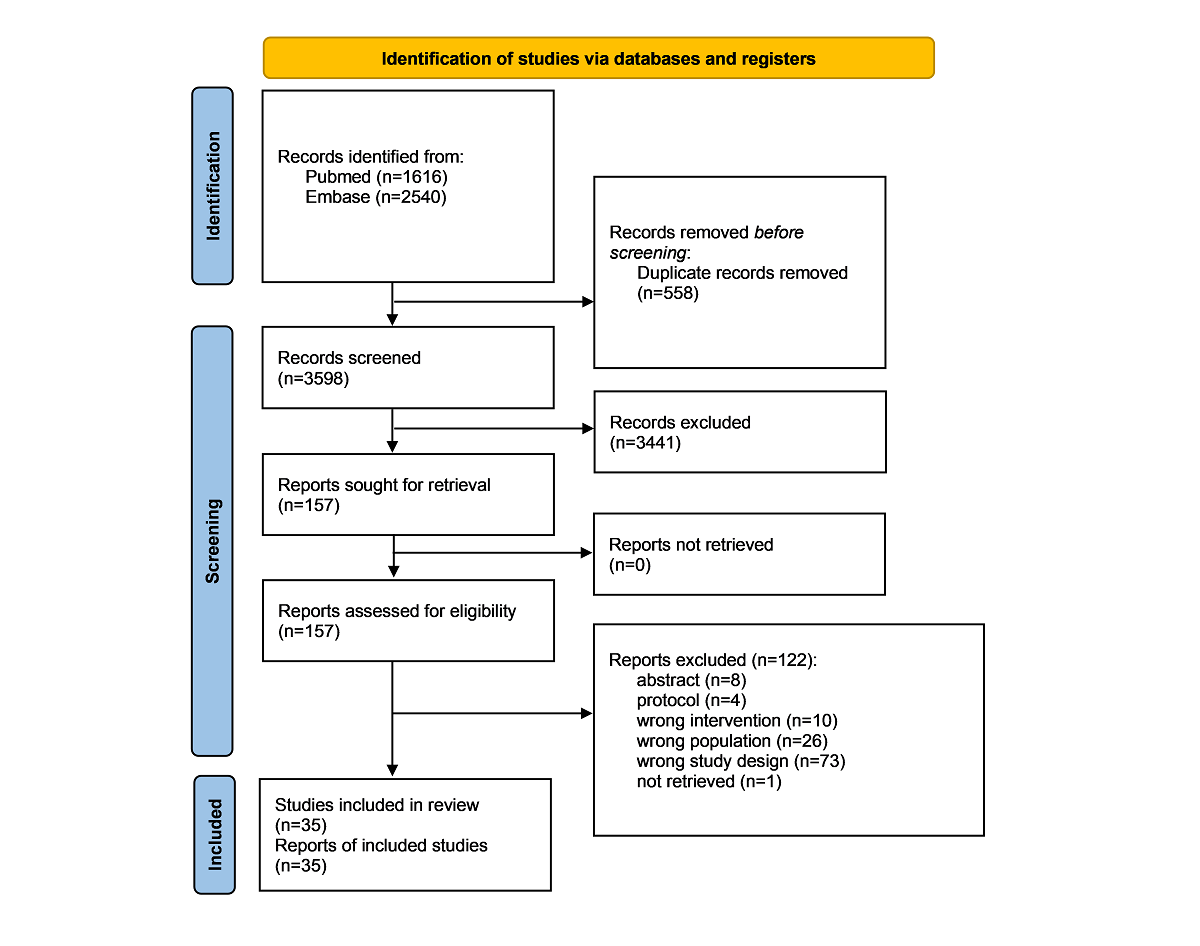
Flow diagram.

After removing duplicates, 3598 records were identified. Of the 157 full texts assessed, 122 SRs were excluded and 35 were included. Figure 1 shows the PRISMA flowchart. The reasons for excluding certain studies after reading their full texts are presented in Multimedia Appendix 2 [13-15,28-59], Table S1.

### Characteristics of Included SRs

Most SRs were published in Europe (n=15), followed by the Americas (n=10), Asia (n=7), and Oceania (n=3). The median publication year was 2021 (minimum 2015, maximum 2022), with 77% (n=27) of SRs published in the last 3 years (2020-2022). Most SRs assessed telerehabilitation (n=29). A few assessed digital self-management (n=4) and teleconsultation, telediagnostics, and telemonitoring (n=2). The most common musculoskeletal disorder investigated was osteoarthritis (eg, hip and knee replacement; n=13) and mixed conditions (eg, chronic musculoskeletal pain; n=14). Twenty-two SRs (63%) reported a meta-analysis. More details are reported in Table 1.

**Table 1:** General characteristics of included studies (n=35).

Characteristics		Studies, n (%)
**Country**		
	China	6 (17)
	Spain	4 (11)
	United Kingdom	4 (11)
	United States	4 (11)
	Australia	3 (9)
	Brazil	3 (9)
	Canada	3 (9)
	Finland	2 (6)
	Germany	2 (6)
	Ireland	1 (3)
	Italy	1 (3)
	Netherlands	1 (3)
	Pakistan	1 (3)
**Years**		
	2020-2022	27 (77)
	2017-2019	7 (20)
	2014-2016	1 (3)
**Conflicts of interest**		
	No	31 (88)
	Not reported	2 (6)
	Reported	2 (6)
**Funding**		
	Not reported	21 (60)
	No profit	11 (31)
	Mixed	3 (9)
**Population**		
	Mixed	14 (40)
	Osteoarthritis	13 (37)
	Other dorsopathies	4 (11)
	Other disorders of the musculoskeletal system and connective tissue	3 (9)
	Autoinflammatory syndromes	1 (3)
**Intervention**		
	Telerehabilitation	29 (83)
	Digital self-management	4 (11)
	Teleconsultation, telediagnostics, monitoring	2 (6)
**Meta-analysis**		
	Yes	22 (63)
	No	13 (27)

### Risk of Bias (Quality) Assessment

**Figure 2:**
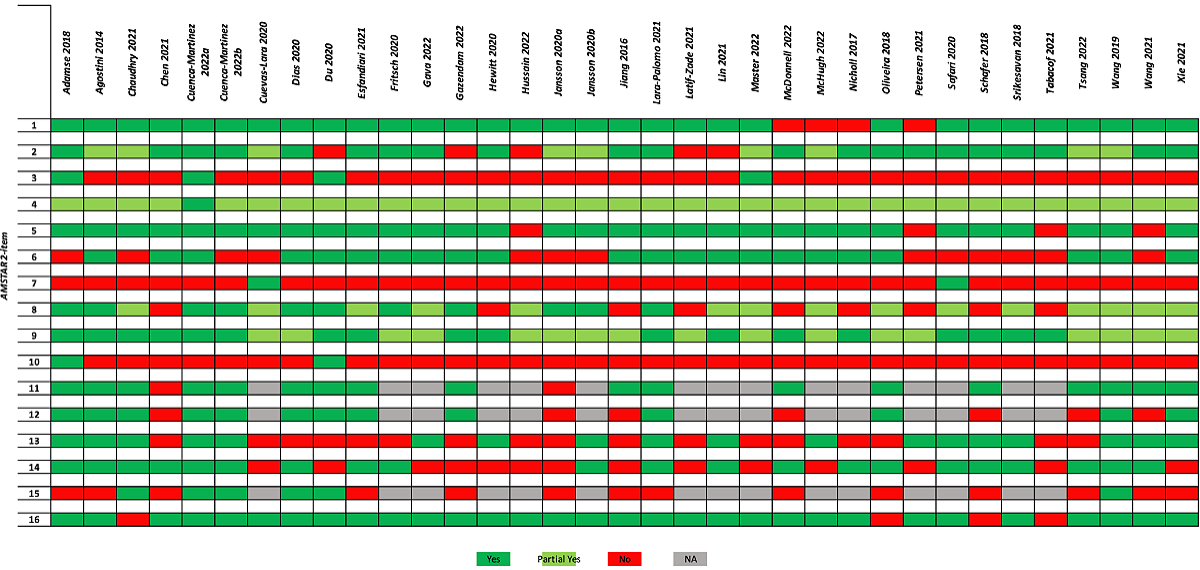
AMSTAR 2 (A Measurement Tool to Assess systematic Reviews 2) summary plot. Item 1: “Did the research questions and inclusion criteria for the review include the components of PICO?”; item 2: “Did the report of the review contain an explicit statement that the review methods were established prior to the conduct of the review and did the report justify any significant deviations from the protocol?”; item 3: “Did the review authors explain their selection of the study designs for inclusion in the review?”; item 4: “Did the review authors use a comprehensive literature search strategy?”; item 5: “Did the review authors perform study selection in duplicate?”; item 6: “Did the review authors perform data extraction in duplicate?”; item 7: “Did the review authors provide a list of excluded studies and justify the exclusions?”; item 8: “Did the review authors describe the included studies in adequate detail?”; item 9: “Did the review authors use a satisfactory technique for assessing the risk of bias RoB in individual studies that were included in the review?”; item 10: “Did the review authors report on the sources of funding for the studies included in the review?”; item 11: “If meta-analysis was performed, did the review authors use appropriate methods for statistical combination of results?”; item 12: “If meta-analysis was performed, did the review authors assess the potential impact of RoB in individual studies on the results of the meta-analysis or other evidence synthesis?”; item 13: “Did the review authors account for RoB in primary studies when interpreting/discussing the results of the review?”; item 14: “Did the review authors provide a satisfactory explanation for, and discussion of, any heterogeneity observed in the results of the review?”; item 15: “If they performed quantitative synthesis did the review authors carry out an adequate investigation of publication bias small study bias and discuss its likely impact on the results of the review?”; item 16: “Did the review authors report any potential sources of conflict of interest, including any funding they received for conducting the review?”.

Methodological quality was critically low in 69% (n=24) of the SRs, low in 29% (n=10), and moderate in 1. Among critical items, 80% of SRs did not report reasons for exclusion (n=28), 48% did not account for risk of bias in individual studies when interpreting or discussing the results of the review (n=17), and 31% did not carry out an adequate investigation of publication bias (eg, small study bias; n=11). A summary plot is shown in Figure 2 [13-15,28-59].

### Outcome Characteristics

**Figure 3:**
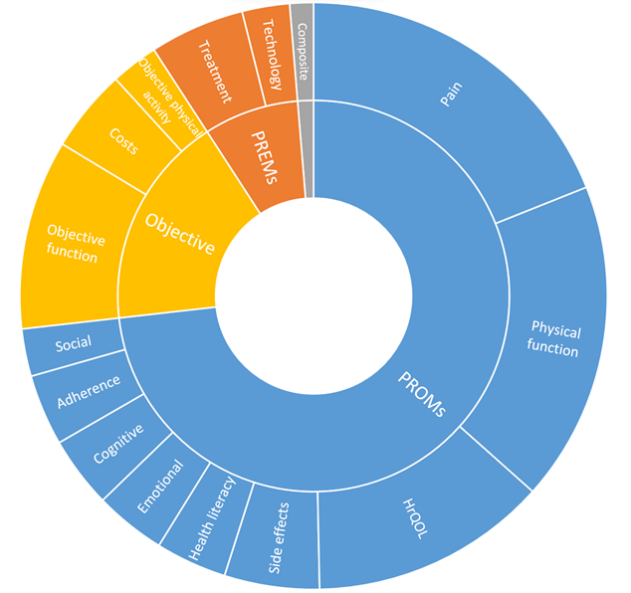
Outcomes reported by reviews. The frequencies of each outcome category reflect the number of reviews addressing them. Inner circles represent the 3 categories defined in the methods: objective outcomes, patient-reported outcome measures (PROMs), and patient-reported experience measures (PREMs); outer circles represent the outcome domains (eg, physical function). HrQoL: health-related quality of life.

Overall, 190 outcomes were collected, among which were 142 PROMs and 15 PREMs; 31 objective outcomes were also collected, including 19 related to physical function, 4 to physical activity (eg, steps per day), and 8 to costs; 2 were composite outcomes (ie, PROMs plus objective measures). The most reported PROMs were related to pain assessment (n=40 in 29 reviews), physical function (n=35 in 27 reviews), and HRQoL (n=20 in 20 reviews). Among PREMs, technology was assessed in 4 reviews (11%) and treatment in 8 reviews (23%). Figure 3 illustrates the outcomes assessed by the reviews in subgroup for PROMs, PREMs, and objective measures.

### Summary of Quantitative Analyses

A total of 23 reviews (66%) reported a meta-analysis for a total of 79 analyses. Among these, 60 meta-analyses were performed on PROMs, 4 on PREMs, 13 on objective outcomes, and 2 on composite outcomes (ie, PROMs plus objective measurements).

Most PROMs were quantitatively analyzed in the following categories: pain assessment (n=23), physical function (n=21), HRQoL (n=8), cognitive function (n=2), emotional function (n=4), health literacy (n=1), and social function (n=1). The 37% (n=22) of meta-analyses assessing PROMs had results favoring telemedicine, 58% (n=35) found no differences between groups, and a few favored controls (n=3). No quantitative analyses for side effects or adherence were found. Meta-analyses of PREMs analyzed only treatment experiences and none showed any difference between groups. All the 13 objective outcomes meta-analyzed were on physical function (eg, balance test, Time Up and Go test, range of motion, 6-minute walking test), mainly favoring telemedicine (n=2) or showing no differences between groups (n=7), while a few (n=4) favored controls. Composite outcomes including PROMs and objective measures were analyzed in 2 meta-analyses; these showed no differences between groups. Overall, reported heterogeneity ranged from 0 to 97 (median *I*^2^ 28.2, IQR 0-67.8)

**Figure 4:**
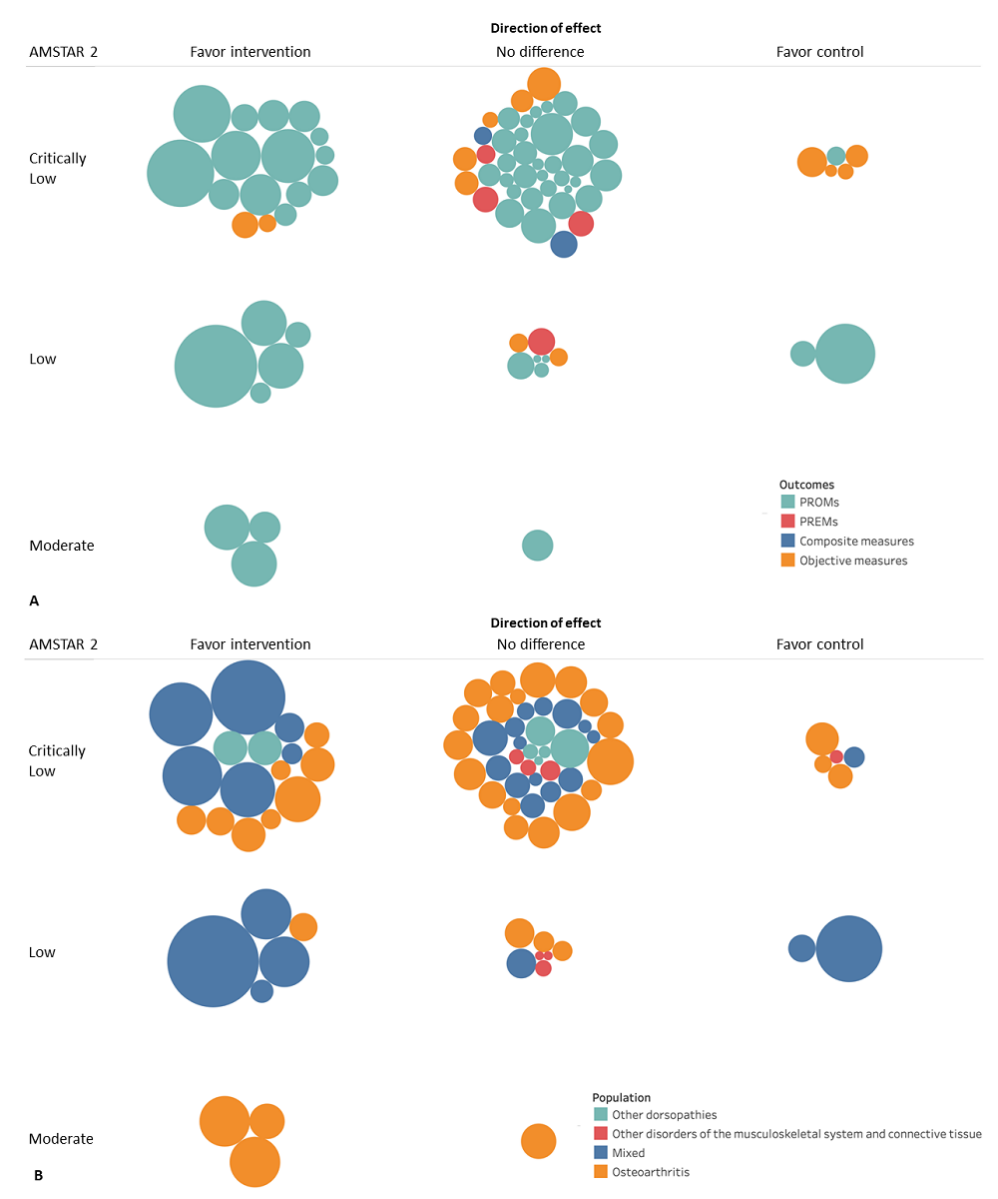
Bubble plot showing directions of effects and AMSTAR 2 (A Measurement Tool to Assess systematic Reviews 2) results by (A) outcomes and (B) type of population. This graphic provides information in three dimensions: (1) in the x-axis, the authors’ conclusions are rated as “beneficial for intervention,” “no effect,” or “beneficial for control” (this is further described in the Data Extraction section); (2) in the y-axis, the quality assessment (AMSTAR 2) is shown; and (3) the bubble size is proportional to the number of participants included in each systematic review. PREM: patient-reported outcome measure; PROM: patient-reported experience measure.

Figure 4 represents, with bubble plots, the direction of results and methodological quality of reviews in subgroups for outcome categories and populations.

### Summary of Qualitative Analyses

Twenty-four SRs reported qualitative results. Overall, 82 PROMs were described in the following areas: pain assessment (n=16), physical function (n=14), HRQoL (n=13), health literacy (n=9), cognitive function (n=8), side effects (n=8), adherence (n=7), emotional function (n=4), and social function (n=3). The SRs reported heterogeneous results in terms of physical function and HRQoL. Most of the SRs reported beneficial results in terms of health literacy and cognitive function in favor of telemedicine intervention, that is, the effects of the experimental intervention were superior or equal to control in all other outcomes. Eight of the 12 PREMs included by the SRs were related to treatment and 4 to technology; 9 of the 21 objective outcomes were related to physical function, 4 to physical activity, and 8 to costs. Considering costs, all SRs showed that telemedicine cost significantly less than in-person visits or usual care. Multimedia Appendix 3 qualitatively describes these outcomes divided by domain of interest.

## Discussion

### Main Findings

This umbrella review summarizes the results from 35 SRs on telemedicine for musculoskeletal disorders, mainly published in the last 3 years (2020-2022) and predominantly conducted in Europe and North America. The type of telemedicine most often assessed was telerehabilitation for mixed chronic musculoskeletal pain and osteoarthritis. Overall, we retrieved many PROMs assessing pain, physical function, and HRQoL, whereas PREMs were less investigated.

According to our results, PROMs are more frequently analyzed than objective measures and PREMs, with most meta-analyses showing an improvement or similar effects compared to any other kind of intervention (eg, in-person treatment, usual care, or waiting list). The same effects were found in different subgroups of populations when visualized by direction of effects. However, some PROMs, such as side effects and adherence, were not quantitatively analyzed. It can be hypothesized that primary studies did not offer useful data to be pooled in a synthesis due to different taxonomies or poor outcome reporting, as well as the presence of outcome nonreporting bias [60,61].

Narrative syntheses on PREMs were reported in few SRs. Superior or equal effects were reported by telemedicine interventions in terms of acceptability of technology (usability, enjoyment, patient experience) and beneficial effects in terms of treatment (patient satisfaction and motivation). Costs were poorly investigated, but it seems that direct and indirect costs were reduced when telemedicine intervention is provided in comparison to in-person visits or usual care.

Overall, SR results should be interpreted with caution considering their methodological quality, which was generally critically low.

### Comparison With Previous Overviews

Our findings are consistent with a previous overview [62] on the use of telemedicine across the 53 member states of the World Health Organization European Region, which showed clear benefits of telemedicine interventions in the screening, diagnosis, management, treatment, and long-term follow-up of many clinically and epidemiologically relevant diseases. Other overviews found that telemedicine delivered in different forms (eg, telerehabilitation, teleconsultation, and telemonitoring) has the potential to improve clinical outcomes in patients with cardiovascular disease [63] and chronic obstructive respiratory diseases [64]; patients who have survived cancer [65]; patients requiring neurorehabilitation [66] or urology care [67]; and patients with diabetes, dyslipidemia, and hypertension [68]. However, there are some areas of intervention that have not yet been covered by telemedicine and have uncertain effectiveness [68]. Nevertheless, in the musculoskeletal field, our umbrella review agrees with a previous study that supported the use of devices, tools, or software applications to facilitate remote rehabilitation and health care in general [69].

### Clinical Implications

Clinicians and stakeholders should consider the adoption of the best available telemedicine technologies to treat patients’ acute and chronic conditions, both in ordinary [70] and extraordinary situations [71]; evidence-based exercise and education [72] can be tailored and delivered remotely, for instance, to increase patients’ compliance to treatment [28,73], reduce withdrawal rates from follow-up (so-called no-show patients), optimize workforce efficiency [74], and sometimes reduce health care costs [75].

Patient-centered care builds on the consideration of individual preference, easier access to treatment, and digital literacy enhancement. However, several barriers still exist in terms of ethical issues, privacy, accessibility, and data security. Telemedicine may help reach people living and working in rural and remote areas [76] with limited medical facilities and personnel. Indeed, patients living in rural areas can have poorer health outcomes in comparison to their urban counterparts [77], highlighting unequal health coverage [78]. An equal use of telemedicine technologies needs countries to invest in effective information policies and communication infrastructures [79]. One example is an Australian Commonwealth government program that aims to expand the Medical Benefit Schedule (MBS) by including telephone or online health consultations to reduce inequalities in favor of rural or remote patients.

### Research Implications

Further efforts should be pursued to standardize collection of PROMs and PREMs in studies evaluating telemedicine. One significant challenge for certain musculoskeletal conditions is the lack of uniformity in outcome measurement across clinical trials; better standardization might help to identify and include PROMs and PREMs in core outcome sets to be measured and reported in all trials of a specific condition [80]. At present, for musculoskeletal disorders, there is still no clear consensus on PROMs as a core outcome set, even though some sets have been developed for Norway and the United Kingdom. Future research is needed to validate these in other countries [81,82].

Considering the lack of evidence on the use of PREMs in the evaluation of telemedicine technologies, they should be used more frequently and consistently before and after interventions. Few SRs reported data regarding costs, which is worth studying in more detail, taking into account the comparative difficulties related to different health care systems [83] and the different means of economic evaluation generally adopted [6,84].

Currently, research is ongoing to identify common core outcome domains from core outcome sets of musculoskeletal conditions [85].

### Strengths and Limitations

To our knowledge, this is the first umbrella review encompassing any kind of telemedicine for different musculoskeletal disorders, including multiple clinical outcomes and costs.

However, some limitations should be mentioned. Only 2 databases were explored and some relevant SRs from other sources (eg, gray literature) may have been missed. Inclusion criteria were focused on SRs of RCTs only. This criterion might have influenced the ratio of PROMs to PREMs, as this may depend on the study type and evidence level [8]. In fact, a previous study [8] investigating the use of PROMs and PREMs in any population with a telemedicine prescription found that the frequency of PREMs decreased with an increasing evidence level (ie, RCTs). Nevertheless, RCTs and reports with the highest quality of evidence should also include information on the usability and acceptance of the technology, expressed as PREMs, in addition to PROMs.

Moreover, we collected and interpreted quantitative effects in the SRs only at the shortest follow-up, limiting generalizability to all time end points. As well, it is possible that the overall positive effects might be biased by the wide range of heterogeneity within the included meta-analyses.

Finally, published taxonomies were used to standardize populations, interventions, and outcomes; however, the categorization of interventions was made difficult by the heterogeneous definition of telemedicine given by the SRs (ie, some used hybrid telemedicine or telemedicine mixed with conventional care, some did not; some used synchronous telemedicine, some did not). Indeed, *telemedicine* is considered an umbrella term for all health care services [1,2].

### Conclusion

Telemedicine for musculoskeletal conditions can provide more accessible health care, with noninferior results in multiple clinical outcomes and no increase in side effects in comparison with more conventional care. The assessment of telemedicine is largely represented by PROMs, reflecting the relevance of patient-centered care. From a cost-effectiveness point of view, future studies should put effort into investigating PREMs, objective measures, and costs, filling the gaps in this promising area.

## Appendix

Multimedia Appendix 1 Search strategy, taxonomy, and methodological quality assessment.

Multimedia Appendix 2 List of included and excluded studies, general characteristics of included systematic reviews, and effect sizes.

Multimedia Appendix 3 Narrative synthesis of patient-reported outcomes and experiences.

Multimedia Appendix 4 PRISMA checklist.

## Abbreviations

AMSTAR 2: A Measurement Tool to Assess systematic Reviews 2
HRQoL: health-related quality of life
ICD-10: International Classification of Diseases, 10th revision
MD: mean difference SMD: standardized mean difference
OR: odds ratio
PICOS: population, intervention, comparison, outcomes, study design
PREM: patient-reported experience measure
PRIOR: Preferred Reporting Items for Overviews of Reviews
PRISMA: Preferred Reporting Items for Systematic Reviews and Meta-Analyses
PROM: patient-reported outcome measure
PROSPERO: Prospective Register of Systematic Reviews
RCT: randomized controlled trial
RR: relative risk
SR: systematic review

## Data Availability

The data sets generated during and/or analyzed during this study are available in the Open Science Framework repository [86].
