# Supplementary material for: Effectiveness of Telemedicine for Musculoskeletal Disorders: Umbrella Review
Source: J Med Internet Res. 2024 Feb 2;26:e50090. doi: 10.2196/50090 (PMC10873802; doi:10.2196/50090)
Supplement: Multimedia Appendix 1 [file jmir_v26i1e50090_app1.docx]

# Multimedia Appendix 1. eMethods

## Table S1 - Search strategy

| **PUBMED** |
| --- |
| ("Orthopedics"[Mesh] OR orthopaedic* [tiab] OR orthopedic* [tiab] OR "musculoskeletal |
| system"[MeSH Terms] OR Musculoskeletal [tiab] OR Musculo-skeletal [tiab] OR “Musculo skeletal”[tiab] OR rehabilitation [MeSH Terms] OR "Physical Therapy Modalities" [MeSH Terms] OR physiotherapy [tiab] or rehabilitation [tiab] or “physical therap*” [tiab]) |
| AND |
| ("Telerehabilitation"[Mesh] OR Telerehabilitation* [tiab] OR tele-rehabilitation* [tiab] OR “telerehabilitation*” [tiab] OR "Telemedicine*"[Mesh] OR telemedicine* [tiab] OR tele-medicine [tiab] OR “tele medicine” [tiab] OR "Remote Consultation"[Mesh] OR “remote consultation*” [tiab] OR "Videoconferencing"[Mesh] OR videoconferenc* [tiab] OR video-conferenc* [tiab] OR “video conferenc*” [tiab] OR "Wearable Electronic Devices"[Mesh] OR wearabl* [tiab] OR “activity sensor*” [tiab] OR monitor* [tiab] OR e-health [tiab] OR ehealth [tiab] OR “electronic health” [tiab] OR m-health [tiab] OR mhealth [tiab] OR “mobile health” [tiab] OR tele-health [tiab] OR “digital health” [tiab] OR “digital medicine” [tiab] OR "Mobile Health Units"[Mesh] OR “health technolog* “[tiab]) |
| AND |
| ("Systematic review"[ptyp] OR "systematic reviews as topic" [MeSH Terms] or "systematic review"[Tiab] OR "meta analys*" [Tiab] OR meta-analys* [Tiab] OR metaanalys* [Tiab]) |
|  |
|  |
| **EMBASE** |
| ('orthopedics'/exp OR 'orthopaedic*':ab,ti OR 'orthopedic*':ab,ti OR 'musculoskeletal system'/exp OR 'musculoskeletal':ab,ti OR 'musculo-skeletal':ab,ti OR 'musculo skeletal':ab,ti OR 'rehabilitation'/exp OR 'rehabilitation' OR 'physiotherapy'/exp OR 'physiotherapy':ab,ti OR 'rehabilitation':ab,ti OR 'physical therap*':ab,ti) |
| AND |
| ('telerehabilitation'/exp OR 'telerehabilitation*':ab,ti OR 'tele-rehabilitation*':ab,ti OR 'tele rehabilitation*':ab,ti OR 'telemedicine'/exp OR 'telemedicine*':ab,ti OR 'tele-medicine':ab,ti OR 'tele medicine':ab,ti OR 'teleconsultation'/exp OR 'remote consultation*':ab,ti OR 'videoconferencing'/exp OR 'videoconferenc*':ab,ti OR 'video-conferenc*':ab,ti OR 'video conferenc*':ab,ti OR 'wearabl*':ab,ti OR 'activity sensor*':ab,ti OR 'monitor*':ab,ti OR 'e-health':ab,ti OR 'ehealth':ab,ti OR 'electronic health':ab,ti OR 'm-health':ab,ti OR 'mhealth':ab,ti OR 'mobile health':ab,ti OR 'telehealth'/exp OR 'tele-health':ab,ti OR 'digital health':ab,ti OR 'digital medicine':ab,ti OR 'health technolog*':ab,ti) |
| AND |
| ('systematic review'/exp OR 'systematic review':ab,ti OR 'meta analys*':ab,ti OR 'meta-analys*':ab,ti OR 'metaanalys*':ab,ti) AND [embase]/lim |

## Table S2 – PROMs and PREMs taxonomy

| PROMs |  |
| --- | --- |
| Pain | Measures the extent to which the illness affects pain. |
| HRQoL | Measures the HRQoL of the respondent. |
| Physical function | Measures the extent to which the illness affects the physical function of the respondent. |
| Social function | Measures the extent to which the illness affects the social function of the respondent. |
| Emotional function | Measures the extent to which the illness affects the emotional function of the respondent. |
| Cognitive function | Measures the extent to which the illness affects the cognitive function and disease perception of the respondent. |
| Health literacy | Measures the respondent’s ability to avoid, alleviate, or live with a disease. |
| Side effects | Measures complaints caused by therapeutic intervention. |
| Adherence | Measures the active role of the patient in the implementation of a therapy. |
|  |  |
| PREMs |  |
| Treatment | Deals with the experience of the medical component of a telemedical intervention, including treatment satisfaction. |
| Technology | Deals with the experience of the technical component of a telemedical intervention. |

Adapted from a previous published taxonomy:

Knapp, A., L. Harst, S. Hager, J. Schmitt and M. Scheibe (2021). "Use of Patient-Reported Outcome Measures and Patient-Reported Experience Measures Within Evaluation Studies of Telemedicine Applications: Systematic Review." J Med Internet Res 23(11): e30042.

## Table S3. Assessing the methodological quality of systematic reviews (AMSTAR) – range of judgements

| **High** |
| --- |
| No or one non-critical weakness: The systematic review provides an accurate and comprehensive summary of the results of the available studies that address the question of interest. |
|  |
| **Moderate** |
| More than one non-critical weakness*: The systematic review has more than one weakness but no critical flaws. This may provide an accurate summary of the results of the available studies included in the review. |
|  |
| **Low** |
| One critical flaw with or without non-critical weaknesses: The review has a critical flaw and may not provide an accurate and comprehensive summary of the available studies that address the question of interest. |
|  |
| **Critically low** |
| More than one critical flaw with or without non-critical weaknesses: The review has more than one critical flaw and should not be relied on to provide an accurate and comprehensive summary of the available studies. |

*Multiple non-critical weaknesses may diminish confidence in the review, and it may be appropriate to move the overall appraisal down from moderate to low confidence.

**AMSTAR 2 critical domains**

- Protocol registered before commencement of the review (item 2)
- Adequacy of the literature search (item 4)
- Justification for excluding individual studies (item 7)
- Risk of bias from individual studies being included in the review (item 9)
- Appropriateness of meta-analytical methods (item 11)
- Consideration of risk of bias when interpreting the results of the review (item 13)
- Assessment of presence and likely impact of publication bias (item 15)

Shea BJ, Grimshaw JM, Wells GA, Boers M, Andersson N, Hamel C, Porter AC, Tugwell P, Moher D, Bouter LM. Development of AMSTAR: a measurement tool to assess the methodological quality of systematic reviews. BMC Med Res Methodol. 2007 Feb 15;7:10. doi: 10.1186/1471-2288-7-10. PMID: 17302989; PMCID: PMC1810543
