# Supplementary material for: Effectiveness of Telemedicine for Musculoskeletal Disorders: Umbrella Review
Source: J Med Internet Res. 2024 Feb 2;26:e50090. doi: 10.2196/50090 (PMC10873802; doi:10.2196/50090)
Supplement: Multimedia Appendix 2 [file jmir_v26i1e50090_app2.docx]

# Multimedia Appendix 2. Results

## Table S1A - List of included studies

| **Authors** | **Title** | **Year** | **Journal** |
| --- | --- | --- | --- |
| **Included studies** | | | |
| Adamse | The effectiveness of exercise-based telemedicine on pain, physical activity and quality of life in the treatment of chronic pain: A systematic review. | 2018 | J Telemed Telecare |
| Agostini | Telerehabilitation and recovery of motor function: a systematic review and meta-analysis. | 2015 | J Telemed Telecare |
| Chaudhry | How Satisfied Are Patients and Surgeons with Telemedicine in Orthopaedic Care During the COVID-19 Pandemic? A Systematic Review and Meta-analysis. | 2021 | Clin Orthop Relat Res |
| Chen | Effects of technology-supported exercise programs on the knee pain, physical function, and quality of life of individuals with knee osteoarthritis and/or chronic knee pain: A systematic review and meta-analysis of randomized controlled trials. | 2021 | J Am Med Inform Assoc |
| Cuenca-Martinez | Implementation of Online Behavior Modification Techniques in the Management of Chronic Musculoskeletal Pain: A Systematic Review and Meta-Analysis. | 2022 | J Clin Med |
| Cuenca-Martinez | Effectiveness of Telematic Behavioral Techniques to Manage Anxiety, Stress and Depressive Symptoms in Patients with Chronic Musculoskeletal Pain: A Systematic Review and Meta-Analysis. | 2022 | Int J Environ Res Public Health |
| Cuevas-Lara | Impact of Game-Based Interventions on Health-Related Outcomes in Hospitalized Older Patients: A Systematic Review. | 2021 | Journal of the American Medical Directors Association |
| Dias | Effectiveness of exercises by telerehabilitation on pain, physical function and quality of life in people with physical disabilities: a systematic review of randomised controlled trials with GRADE recommendations. | 2021 | Br J Sports Med |
| Du | The efficacy of e-health in the self-management of chronic low back pain: A meta analysis. | 2020 | Int J Nurs Stud |
| Esfandiari | Telehealth interventions for mobility after lower limb loss: A systematic review and meta-analysis of randomized controlled trials. | 2022 | Prosthetics and Orthotics International |
| Fritsch | Effects of using text message interventions for the management of musculoskeletal pain: a systematic review. | 2020 | Pain |
| Gava | Effectiveness of physical therapy given by telerehabilitation on pain and disability of individuals with shoulder pain: A systematic review. | 2022 | Clin Rehabil |
| Gazendam | Virtual reality rehabilitation following total knee arthroplasty: a systematic review and meta-analysis of randomized controlled trials. | 2022 | Knee Surg Sports Traumatol Arthrosc |
| Hewitt | The Effectiveness of Digital Health Interventions in the Management of Musculoskeletal Conditions: Systematic Literature Review. | 2020 | J Med Internet Res |
| Hussain | Digital technologies in management of chronic pain - a systematic review. | 2022 | J Pak Med Assoc |
| Jansson | Computer- and Telephone-Delivered Interventions on Patient Outcomes and Resource Utilization in Patients With Orthopaedic Conditions: A Systematic Review and Narrative Synthesis. | 2020b | Orthop Nurs |
| Jansson | The effects and safety of telerehabilitation in patients with lower-limb joint replacement: A systematic review and narrative synthesis. | 2020a | J Telemed Telecare |
| Jiang | The comparison of telerehabilitation and face-to-face rehabilitation after total knee arthroplasty: A systematic review and meta-analysis. | 2018 | J Telemed Telecare |
| Lara-Palomo | Efficacy of e-Health Interventions in Patients with Chronic Low-Back Pain: A Systematic Review with Meta-Analysis. | 2022 | Telemed J E Health |
| Latif-Zade | Systematic Review Shows Tele-Rehabilitation Might Achieve Comparable Results to Office-Based Rehabilitation for Decreasing Pain in Patients with Knee Osteoarthritis. | 2021 | Medicina (Kaunas) |
| Lin | Effect of Virtual Reality on Functional Ankle Instability Rehabilitation: A Systematic Review. | 2021 | J Healthc Eng |
| Master | Effects of physical activity interventions using wearables to improve objectively-measured and patient-reported outcomes in adults following orthopaedic surgical procedures: A systematic review. | 2022 | PLoS One |
| McDonnell | The efficacy of remote virtual care in comparison to traditional clinical visits for elective orthopaedic patients: A meta-analysis of prospective randomised controlled trials. | 2022 | Surgeon |
| McHugh | Effectiveness of remote exercise programs in reducing pain for patients with knee osteoarthritis: A systematic review of randomized trials. | 2022 | Osteoarthritis and Cartilage Open |
| Nicholl | Digital Support Interventions for the Self-Management of Low Back Pain: A Systematic Review. | 2017 | J Med Internet Res |
| Oliveira | Physical Activity-Based Interventions Using Electronic Feedback May Be Ineffective in Reducing Pain and Disability in Patients With Chronic Musculoskeletal Pain: A Systematic Review With Meta-Analysis. | 2018 | Archives of Physical Medicine and Rehabilitation |
| Petersen | A systematic review about telemedicine in orthopedics. | 2021 | Arch Orthop Trauma Surg |
| Safari | Digital Self-Management Interventions for People With Osteoarthritis: Systematic Review With Meta-Analysis. | 2020 | J Med Internet Res |
| Schäfer | The Efficacy of Electronic Health-Supported Home Exercise Interventions for Patients With Osteoarthritis of the Knee: Systematic Review. | 2018 | J Med Internet Res |
| Srikesavan | Web-based rehabilitation interventions for people with rheumatoid arthritis: A systematic review. | 2019 | J Telemed Telecare |
| Tabacof | Telehealth treatment for nonspecific low back pain: A review of the current state in mobile health. | 2021 | PM and R |
| Tsang | The effectiveness of telerehabilitation in patients after total knee replacement: A systematic review and meta-analysis of randomized controlled trials. | 2022 | J Telemed Telecare |
| Wang | The effectiveness of internet-based telerehabilitation among patients after total joint arthroplasty: A systematic review and meta-analysis of randomised controlled trials. | 2021 | J Telemed Telecare |
| Wang | Technology-assisted rehabilitation following total knee or hip replacement for people with osteoarthritis: a systematic review and meta-analysis. | 2019 | BMC Musculoskelet Disord |
| Xie | Effect of Internet-Based Rehabilitation Programs on Improvement of Pain and Physical Function in Patients with Knee Osteoarthritis: Systematic Review and Meta-analysis of Randomized Controlled Trials. | 2021 | J Med Internet Res |

## Table S1B - List of excluded studies

| **Authors** | **Title** | **Year** | **Journal** | **Reason of exclusion** | |
| --- | --- | --- | --- | --- | --- |
| **Excluded studies** | | | | |  |
| Medical Advisory Secretariat | Physiotherapy rehabilitation after total knee or hip replacement: an evidence-based analysis. | 2005 | Ont Health Technol Assess Ser | intervention | |
| Ajrawat | The Use of Telehealth for Orthopedic Consultations and Assessments: A Systematic Review. | 2021 | Orthopedics | study design | |
| Alarcón-Aldana | Upper Limb Physical Rehabilitation Using Serious Videogames and Motion Capture Systems: A Systematic Review. | 2020 | Sensors (Basel) | study design | |
| Alexandre | Telerehabilitation versus conventional face-to-face land-based exercises following hip or knee arthroplasty. | 2021 | Cochrane Database of Systematic Reviews | protocol | |
| Allet | Wearable systems for monitoring mobility-related activities in chronic disease: a systematic review. | 2010 | Sensors (Basel) | population | |
| Almeida | Telerehabilitation for acute, subacute and chronic low back pain. | 2020 | Cochrane Database of Systematic Reviews | protocol | |
| Ambrens | Effect of eHealth-delivered exercise programmes on balance in people aged 65 years and over living in the community: a systematic review and meta-analysis of randomised controlled trials. | 2022 | BMJ Open | population | |
| Amin | Is physiotherapy an underused approach to prevent surgery in selective musculoskeletal disorders? | 2021 | Bangladesh Journal of Medical Science | study design | |
| Badawy | Habit Strength, Medication Adherence, and Habit-Based Mobile Health Interventions across Chronic Medical Conditions: Systematic Review. | 2020 | Journal of Medical Internet Research | population | |
| Bahadori | A review of current use of commercial wearable technology and smartphone apps with application in monitoring individuals following total hip replacement surgery. | 2020 | J Med Eng Technol | study design | |
| Berger-Groch | The Use of Mobile Applications for the Diagnosis and Treatment of Tumors in Orthopaedic Oncology - a Systematic Review. | 2021 | J Med Syst | study design | |
| Berton | Virtual Reality, Augmented Reality, Gamification, and Telerehabilitation: Psychological Impact on Orthopedic Patients' Rehabilitation. | 2020 | J Clin Med | study design | |
| Booth | The Effectiveness of Interventions and Intervention Components for Increasing Physical Activity and Reducing Sedentary Behaviour in People With Persistent Musculoskeletal Pain: A Systematic Review and Meta-Analysis. | 2022 | J Pain | intervention | |
| Bordeleau | The Use of Virtual Reality in Back Pain Rehabilitation: A Systematic Review and Meta-Analysis. | 2022 | Journal of Pain | study design | |
| Brainard | A systematic review of health service interventions to reduce use of unplanned health care in rural areas. | 2016 | J Eval Clin Pract | population | |
| Bright | What Is the Proportion of Studies Reporting Patient and Practitioner Satisfaction with Software Support Tools Used in the Management of Knee Pain and Is This Related to Sample Size, Effect Size, and Journal Impact Factor? | 2018 | Telemed J E Health | study design | |
| Carnevale | Wearable systems for shoulder kinematics assessment: a systematic review. | 2019 | BMC Musculoskelet Disord | study design | |
| Carpino | Assessing Effectiveness and Costs in Robot-Mediated Lower Limbs Rehabilitation: A Meta-Analysis and State of the Art. | 2018 | J Healthc Eng | study design | |
| Chadwell | Technology for monitoring everyday prosthesis use: A systematic review. | 2020 | Journal of NeuroEngineering and Rehabilitation | study design | |
| Chakravorty | The role of wearable devices and objective gait analysis for the assessment and monitoring of patients with lumbar spinal stenosis: systematic review. | 2019 | BMC Musculoskelet Disord | study design | |
| Chan | Wearable Activity Monitors in Home Based Exercise Therapy for Patients with Intermittent Claudication: A Systematic Review. | 2021 | Eur J Vasc Endovasc Surg | population | |
| Cheung | Review of accelerometry for determining daily activity among elderly patients. | 2011 | Archives of Physical Medicine and Rehabilitation | population | |
| Choi | mHealth technologies for osteoarthritis self-management and treatment: A systematic review. | 2019 | Health Informatics J | study design | |
| Chua | The Willingness to Pay for Telemedicine Among Patients With Chronic Diseases: Systematic Review. | 2022 | Journal of Medical Internet Research | population | |
| Claes | Factors associated with better treatment outcome of physical therapy interventions after shoulder arthroplasty: A systematic review. | 2022 | Clin Rehabil | study design | |
| Coda | A Review of Online Rehabilitation Protocols Designated for Rotator Cuff Repairs. | 2020 | Arthroscopy, Sports Medicine, and Rehabilitation | intervention | |
| Coenen | Integrated care programmes for sport and work participation, performance of physical activities and quality of life among orthopaedic surgery patients: a systematic review with meta-analysis. | 2020 | BMJ Open Sport Exerc Med | study design | |
| Corso | Are Nonpharmacologic Interventions Delivered Through Synchronous Telehealth as Effective and Safe as In-Person Interventions for the Management of Patients With Nonacute Musculoskeletal Conditions? A Systematic Rapid Review. | 2022 | Arch Phys Med Rehabil | study design | |
| Cottrell | Real-time telerehabilitation for the treatment of musculoskeletal conditions is effective and comparable to standard practice: a systematic review and meta-analysis. | 2017 | Clin Rehabil | study design | |
| Davergne | Use of Wearable Activity Trackers to Improve Physical Activity Behavior in Patients With Rheumatic and Musculoskeletal Diseases: A Systematic Review and Meta-Analysis. | 2019 | Arthritis Care Res (Hoboken) | intervention | |
| Davey | Virtual Fracture Clinics in Orthopaedic Surgery - A Systematic Review of Current Evidence. | 2020 | Injury | study design | |
| De La Cruz Monroy | The Use of Smartphone Applications (Apps) for Enhancing Communication With Surgical Patients: A Systematic Review of the Literature. | 2019 | Surg Innov | population | |
| de Leeuwerk | The effectiveness of physical activity interventions using activity trackers during or after inpatient care: a systematic review and meta-analysis of randomized controlled trials. | 2022 | Int J Behav Nutr Phys Act | population | |
| Debon | Mobile health applications for chronic diseases: A systematic review of features for lifestyle improvement. | 2019 | Diabetes Metab Syndr | population | |
| Dellifraine | Home-based telehealth: a review and meta-analysis. | 2008 | J Telemed Telecare | population | |
| Devan | Evaluation of Self-Management Support Functions in Apps for People With Persistent Pain: Systematic Review. | 2019 | JMIR Mhealth Uhealth | intervention | |
| Fahey | Telemedicine in Orthopedic Surgery: A Systematic Review of Current Evidence. | 2022 | Telemed J E Health | study design | |
| Fan | The effectiveness of exercise based digital health interventions (requiring internet) in management of hip and knee osteoarthritis: a systematic review and meta-analysis. | 2022 | Osteoarthritis and Cartilage | abstract | |
| Fandim | Telerehabilitation for neck pain. | 2021 | Cochrane Database of Systematic Reviews | protocol | |
| Fatoye | POSC71 Economic Evaluations of Digital Health Interventions for the Management of Musculoskeletal Disorders: A Systematic Review. | 2022 | Value in Health | abstract | |
| Fenerty | The effect of reminder systems on patients' adherence to treatment. | 2012 | Patient Preference and Adherence | population | |
| Flodgren | Interactive telemedicine: effects on professional practice and health care outcomes. | 2015 | Cochrane Database Syst Rev | population | |
| Flumignan | What do Cochrane systematic reviews say about telemedicine for healthcare? | 2019 | Sao Paulo Medical Journal | study design | |
| Fraser | Use of telehealth for health care of Indigenous peoples with chronic conditions: a systematic review. | 2017 | Rural Remote Health | population | |
| Gaikwad | The role of home-based information and communications technology interventions in chronic disease management: A systematic literature review. | 2009 | Health Informatics Journal | study design | |
| Gao | Video visits can be both efficient and effective: A systematic review. | 2017 | Journal of General Internal Medicine | abstract | |
| Garcia-Lizana | New technologies for chronic disease management and control: a systematic review. | 2007 | J Telemed Telecare | population | |
| Gilbert | What is the effect of communication technology on the work of being a patient in orthopaedics? A systematic review. | 2020 | Physiotherapy (United Kingdom) | abstract | |
| Gilbert | What is the patient acceptability of real time 1:1 videoconferencing in an orthopaedics setting? A systematic review. | 2018 | Physiotherapy | study design | |
| Gilbert | Use of virtual consultations in an orthopaedic rehabilitation setting: how do changes in the work of being a patient influence patient preferences? A systematic review and qualitative synthesis. | 2020 | BMJ Open | study design | |
| Grigorovich | A systematic review of economic analyses of home-based telerehabilitation. | 2021 | Disabil Rehabil | population | |
| Grona | Use of videoconferencing for physical therapy in people with musculoskeletal conditions: A systematic review. | 2018 | J Telemed Telecare | study design | |
| Haider | Telemedicine in orthopaedics during COVID-19 and beyond: A systematic review. | 2022 | J Telemed Telecare | study design | |
| Hailey | Evidence of benefit from telerehabilitation in routine care: a systematic review. | 2011 | J Telemed Telecare | study design | |
| Halai | CORR Insights¬Æ: How Satisfied Are Patients and Surgeons with Telemedicine in Orthopaedic Care During the COVID-19 Pandemic? A Systematic Review and Meta-analysis. | 2021 | Clin Orthop Relat Res | study design | |
| Hall | Mobile text messaging for health: A systematic review of reviews. | 2015 | Annu. Rev. Public Health | study design | |
| Harkey | Patient Satisfaction with Telehealth in Rural Settings: A Systematic Review. | 2020 | Int J Telerehabil | study design | |
| Hosseiniravandi | Home-based telerehabilitation software systems for remote supervising: a systematic review. | 2020 | Int J Technol Assess Health Care | study design | |
| Irani | Systematic Review of Technology-Based Interventions Targeting Chronically Ill Adults and Their Caregivers. | 2020 | West J Nurs Res | population | |
| Jakob | Factors Influencing Adherence to mHealth Apps for Prevention or Management of Noncommunicable Diseases: Systematic Review. | 2022 | J Med Internet Res | population | |
| Jayakody | Effectiveness of interventions utilising telephone follow up in reducing hospital readmission within 30 days for individuals with chronic disease: a systematic review. | 2016 | BMC Health Serv Res | population | |
| Jennett | The socio-economic impact of telehealth: a systematic review. | 2003 | J Telemed Telecare | study design | |
| Jimenez-Moreno | Measuring Habitual Physical Activity in Neuromuscular Disorders: A Systematic Review. | 2017 | Journal of Neuromuscular Diseases | study design | |
| Jonker | Feasibility of Perioperative eHealth Interventions for Older Surgical Patients: A Systematic Review. | 2020 | J Am Med Dir Assoc | study design | |
| Kairy | A systematic review of clinical outcomes, clinical process, healthcare utilization and costs associated with telerehabilitation. | 2009 | Disabil Rehabil | study design | |
| Keane | Integrated interventions to reduce pressure on acute hospitals: A systematic umbrella review. | 2019 | BMJ Evidence-Based Medicine | study design | |
| Keogh | Assessing the usability of wearable devices to measure gait and physical activity in chronic conditions: a systematic review. | 2021 | Journal of NeuroEngineering and Rehabilitation | study design | |
| Laverdi√®re | Augmented reality in orthopaedics: a systematic review and a window on future possibilities. | 2019 | Bone Joint J | study design | |
| Lee | Substantiating Clinical Effectiveness and Potential Barriers to the Widespread Implementation of Spinal Cord Injury Telerehabilitation: A Systematic Review and Qualitative Synthesis of Randomized Trials in the Recent Past Decade. | 2021 | Telemed Rep | study design | |
| Lewkowicz | Digital Therapeutic Care and Decision Support Interventions for People With Low Back Pain: Systematic Review. | 2021 | JMIR Rehabil Assist Technol | study design | |
| Longoni | Smartphone applications validated for joint angle measurement: a systematic review. | 2019 | Int J Rehabil Res | intervention | |
| Lor | Visualizations Integrated Into Consumer Health Technologies Support Self-management of Chronic Diseases: A Systematic Review. | 2020 | Comput Inform Nurs | population | |
| Lu | Use of Short Message Service and Smartphone Applications in the Management of Surgical Patients: A Systematic Review. | 2018 | Telemed J E Health | population | |
| Machado | Smartphone apps for the self-management of¬†low back pain: A systematic review. | 2016 | Best Pract Res Clin Rheumatol | intervention | |
| Mahmoud | Usability of Telemedicine in Physical Therapy Rehabilitation: Systematic review. | 2021 | JMIR Rehabil Assist Technol | study design | |
| Marmor | Use of Wearable Technology to Measure Activity in Orthopaedic Trauma Patients: A Systematic Review. | 2022 | Indian J Orthop | study design | |
| Marques | Effectiveness of remote care interventions: a systematic review informing the 2022 EULAR Points to Consider for remote care in rheumatic and musculoskeletal diseases. | 2022 | RMD Open | study design | |
| McKeon | Expanding Role of Technology in Rehabilitation After Lower-Extremity Joint Replacement: A Systematic Review. | 2021 | JBJS Rev | study design | |
| Mehta | Home-based physiotherapy in patients following hip fracture surgery: A systematic review and meta-analysis of randomized controlled trials. | 2011 | Physiotherapy (United Kingdom) | intervention | |
| Meijer | Systematic Review on the Effects of Serious Games and Wearable Technology Used in Rehabilitation of Patients With Traumatic Bone and Soft Tissue Injuries. | 2018 | Arch Phys Med Rehabil | study design | |
| Meirte | Electronic patient reported outcome measures in rehabilitation: A systematic review on the use and development of digital questionnaires. | 2018 | Wound Repair and Regeneration | abstract | |
| Melian | Teleconsultation in orthopaedic surgery: A systematic review and meta-analysis of patient and physician experiences. | 2022 | J Telemed Telecare | study design | |
| Moral-Munoz | Smartphone-based systems for physical rehabilitation applications: A systematic review. | 2021 | Assist Technol | study design | |
| Najm | Mobile Health Apps for Self-Management of Rheumatic and Musculoskeletal Diseases: Systematic Literature Review. | 2019 | JMIR Mhealth Uhealth | study design | |
| Najm | The development process of mobile health applications for self-management in patients with rheumatic and musculoskeletal diseases is heterogeneous and often incomplete: Results of a systematic literature review. | 2018 | Annals of the Rheumatic Diseases | abstract | |
| Ni | Current status and future perspectives regarding telemedicine in the management of chronic noncancer pain (CNCP): A systematic review of the literature. | 2013 | Journal of the American Geriatrics Society | study design | |
| Nussbaum | Systematic Review of Mobile Health Applications in Rehabilitation. | 2019 | Arch Phys Med Rehabil | study design | |
| Onose | Mobile Mechatronic/Robotic Orthotic Devices to Assist-Rehabilitate Neuromotor Impairments in the Upper Limb: A Systematic and Synthetic Review. | 2018 | Front Neurosci | study design | |
| Parker | Electronic, mobile and telehealth tools for vulnerable patients with chronic disease: a systematic review and realist synthesis. | 2018 | BMJ Open | study design | |
| Pastora-Bernal | Evidence of Benefit of Telerehabitation After Orthopedic Surgery: A Systematic Review. | 2017 | J Med Internet Res | study design | |
| Patel | The usability of post-operative mobile health applications: A systematic review. | 2020 | British Journal of Surgery | study design | |
| Pavey | The clinical effectiveness and costeffectiveness of exercise referral schemes: A systematic review and economic evaluation. | 2011 | Health Technology Assessment | intervention | |
| Piche | Physical Examination of the Spine Using Telemedicine: A Systematic Review. | 2021 | Global Spine Journal | study design | |
| Piga | Telemedicine for patients with rheumatic diseases: Systematic review and proposal for research agenda. | 2017 | Semin Arthritis Rheum | study design | |
| Robinson | Digital technology to support lifestyle and health behaviour changes in surgical patients: systematic review. | 2021 | BJS Open | study design | |
| Robinson | Digital and Mobile Technologies to Promote Physical Health Behavior Change and Provide Psychological Support for Patients Undergoing Elective Surgery: Meta-Ethnography and Systematic Review. | 2020 | JMIR Mhealth Uhealth | study design | |
| Rogante | Ten years of telerehabilitation: A literature overview of technologies and clinical applications. | 2010 | NeuroRehabilitation | study design | |
| Rush | The efficacy of telehealth delivered educational approaches for patients with chronic diseases: A systematic review. | 2018 | Patient Educ Couns | population | |
| Sadiq | Role of tele-rehabilitation in patients following total hip replacement: Systematic review of clinical trials. | 2022 | J Pak Med Assoc | study design | |
| Saito | Effectiveness and feasibility of home-based telerehabilitation for community-dwelling elderly people in Southeast Asian countries and regions: a systematic review. | 2021 | Aging Clin Exp Res | population | |
| Salerno | Point-of-Care Teleultrasound: A Systematic Review. | 2020 | Telemed J E Health | study design | |
| Samoocha | Effectiveness of web-based interventions on patient empowerment: a systematic review and meta-analysis. | 2010 | J Med Internet Res | population | |
| Saragiotto | Telerehabilitation for hip or knee osteoarthritis. | 2020 | Cochrane Database of Systematic Reviews | protocol | |
| Sardi | A systematic review of gamification in e-Health. | 2017 | J Biomed Inform | study design | |
| Sekhon | Effectiveness of web-based and mobile health interventions designed to enhance adherence to physical activity for people with inflammatory arthritis: A systematic review. | 2021 | Rheumatology Advances in Practice | study design | |
| Seron | Effectiveness of Telerehabilitation in Physical Therapy: A Rapid Overview | 2021 | Phys Ther | study design | |
| Shukla | Role of telerehabilitation in patients following total knee arthroplasty: Evidence from a systematic literature review and meta-analysis. | 2017 | J Telemed Telecare | study design | |
| Siebra | Mobile health support for motor disability individuals: A review focused on holistic assessment and interventions. | 2020 | Technology and Disability | study design | |
| Svendsen | Barriers and facilitators to patient uptake and utilisation of digital interventions for the self-management of low back pain: a systematic review of qualitative studies. | 2020 | BMJ Open | study design | |
| Turnbull | Health Equity in the Effectiveness of Web-Based Health Interventions for the Self-Care of People With Chronic Health Conditions: Systematic Review. | 2020 | J Med Internet Res | study design | |
| Vaikuntharajan | Telephone-Delivered Physiotherapy Interventions Improve Physical Function for Adults With a Chronic Condition: A Systematic Review and Meta-analysis. | 2022 | Arch Phys Med Rehabil | population | |
| van der Meij | The Effect of Perioperative E-Health Interventions on the Postoperative Course: A Systematic Review of Randomised and Non-Randomised Controlled Trials. | 2016 | PLoS One | study design | |
| van Egmond | Effectiveness of physiotherapy with telerehabilitation in surgical patients: a systematic review and meta-analysis. | 2018 | Physiotherapy | population | |
| Van Remoortel | Validity of activity monitors in health and chronic disease: a systematic review. | 2012 | International Journal of Behavioral Nutrition and Physical Activity | study design | |
| Wallace | A systematic review of smartphone applications for chronic pain available for download in the United States. | 2014 | J Opioid Manag | not retrieved | |
| Wang | Digital disruptive technology for rehabilitation following elective surgery for low back pain, knee and hip osteoarthritis: A systematic review and meta-analysis. | 2018 | Arthritis and Rheumatology | abstract | |
| Wang | Technology-assisted rehabilitation following total knee or hip replacement for people with osteoarthritis: a systematic review and meta analysis. | 2019 | Osteoarthritis and Cartilage | abstract | |
| Weber | How Commercially Available Virtual Reality-Based Interventions Are Delivered and Reported in Gait, Posture, and Balance Rehabilitation: A Systematic Review. | 2020 | Phys Ther | study design | |
| Wiegel | Adherence to telemonitoring by electronic patient-reported outcome measures in patients with chronic diseases: A systematic review. | 2021 | International Journal of Environmental Research and Public Health | study design | |
| Willett | Effectiveness of behaviour change techniques in phys-iotherapy interventions to promote physical activity adherence in patients with osteoarthritis: A systematic review. | 2017 | Physiotherapy (United Kingdom) | intervention | |
| Yadav | Utilising Digital Health Technology to Support Patient-Healthcare Provider Communication in Fragility Fracture Recovery: Systematic Review and Meta-Analysis. | 2019 | Int J Environ Res Public Health | study design | |
| Zischke | The utility of physiotherapy assessments delivered by telehealth: A systematic review. | 2021 | J Glob Health | study design | |

## Table S2 – General characteristics of included systematic reviews

| **ID** | **Author** | **Year** | **Country** | **Conflict of interest** | **Sources of funding** | **N. of included trials** | **Population** | **Intervention** |
| --- | --- | --- | --- | --- | --- | --- | --- | --- |
| 1 | Adamse | 2018 | Netherlands | No | Not reported | 16 | Mixed | Exercise based telemedicine |
| 2 | Agostini | 2015 | Italy | No | Not reported | 12 | Osteoarthritis | Telerehabilitation |
| 3 | Chaudhry | 2021 | Canada | No | Not reported | 8 | Mixed | Live video-based consult |
| 4 | Chen | 2021 | Hong Kong | No | No profit | 12 | Osteoarthritis | Technology-supported exercise programs |
| 5 | Cuenca-Martinez | 2022a | Spain | No | Not reported | 58 | Mixed | Online behavioral modification techniques (bmt) |
| 6 | Cuenca-Martinez | 2022b | Spain | No | Not reported | 41 | Mixed | Online behavioral modification techniques (bmt) |
| 7 | Cuevas-Lara | 2021 | Spain | No | Not reported | 4 | Mixed | Serious-game programs using Nintendo Wi |
| 8 | Dias | 2021 | Brasil | No | No profit | 48 | Mixed | Telerehabilitation |
| 9 | Du | 2020 | China | No | Mixed | 8 | Other dorsopathies | E-Health based self-management programs |
| 10 | Esfandiari | 2022 | Canada | No | Not reported | 6 | Other disorders of the musculoskeletal system and connective tissue | Telehealth |
| 11 | Fritsch | 2020 | Australia | No | Not reported | 11 | Mixed | Text messages |
| 12 | Gava | 2022 | Brasil | No | No profit | 6 | Other disorders of the musculoskeletal system and connective tissue | Telerehabilitation |
| 13 | Gazendam | 2022 | Canada | One author | Not reported | 9 | Osteoarthritis | VR rehabilitation |
| 14 | Hewitt | 2020 | United Kingdom | No | Not reported | 19 | Mixed | Digital health interventions |
| 15 | Hussain | 2022 | Pakistan | No | Not reported | 33 | Mixed | Health technologies |
| 16 | Jansson | 2020 | Finland | No | No profit | 9 | Osteoarthritis | Telerehabilitation |
| 17 | Jansson | 2020_computer | Finland | No | No profit | 6 | Mixed | Telerehabilitation + telemedicine |
| 18 | Jiang | 2018 | China | No | Not reported | 4 | Osteoarthritis | Telerehabilitation |
| 19 | Lara-Palomo | 2022 | Spain | No | No profit | 9 | Other dorsopathies | E-health |
| 20 | Latif-Zade | 2021 | United States | No | Not reported | 3 | Osteoarthritis | Telerehabilitation |
| 21 | Lin | 2021 | China | No | Mixed | 5 | Other disorders of the musculoskeletal system and connective tissue | Virtual reality |
| 22 | Master | 2022 | United States | No | Not reported | 6 | Mixed | Wereable device |
| 23 | McDonnell | 2022 | Ireland | No | Not reported | 11 | Mixed | Videoconferencing to conduct remote care/consultation c |
| 24 | McHugh | 2022 | United States | No | No profit | 11 | Osteoarthritis | Remote exercise programs with or withour in person |
| 25 | Nicholl | 2017 | United Kingdom | No | No profit | 9 | Other dorsopathies | Interactive digital interventions to support self-managemen |
| 26 | Oliveira | 2018 | Brasil | Not reported | Not reported | 4 | Mixed | Physical activityebased interventions using electronic feedback |
| 27 | Petersen | 2021 | Germany | No | Not reported | 14 | Mixed | Videoconsultation (telemedicine) |
| 28 | Safari | 2020 | United Kingdom | No | No profit | 8 | Osteoarthritis | Digital-based structured self-management |
| 29 | Schafer | 2018 | Germany | No | Not reported | 7 | Osteoarthritis | Electronic health–supported home exercise |
| 30 | Srikesavan | 2019 | United Kingdom | No | No profit | 6 | Autoinflammatory syndromes | Web-based rehabilitation interventions |
| 31 | Tabacof | 2021 | United States | Not reported | Not reported | 7 | Other dorsopathies | Mobile health (mhealth) |
| 32 | Tsang | 2022 | Hong Kong | No | Not reported | 11 | Osteoarthritis | Telerehabilitation |
| 33 | Wang | 2021 | Australia | No | Not reported | 11 | Osteoarthritis | Internet-based tele- rehabilitation |
| 34 | Wang | 2019BMC | Australia | One author | No profit | 21 | Osteoarthritis | Telerehabilitation, game- or web-based therapy |
| 35 | Xie | 2021 | China | No | Mixed | 4 | Osteoarthritis | Internet-based intervention |

Table S3. Effect sizes of SRs with meta-analysis reporting quantitative synthesis

| **Author year** | **Intervention** | **Control** | **N° of included studies in meta-analysis** | **N° participants** | **Outcome** | **Scale** | **Effect size (95% CI)** | **Direction of effect** | **I2** | **AMSTAR II** |
| --- | --- | --- | --- | --- | --- | --- | --- | --- | --- | --- |
| **Mixed population** | | | | | | | | | | |
| Adamse 2018 | telerehabilitation | no intervention-usual care | 6 | 1925 | pain | 0-10 scale | MD -0.57 (-0.81; -0.34) | favour intervention | 0 | Critically Low |
| Adamse 2018 | telerehabilitation | no intervention-usual care | 2 | 107 | pain | 0-10 scale | MD -0.08 (-0.41; 0.26) | no difference | 0 | Critically Low |
| Adamse 2018 | telerehabilitation | no intervention-usual care | 8 | 2263 | physical function | composite | SMD -0.20 (-0.29; -0.12) | favour intervention | 0 | Critically Low |
| Adamse 2018 | telerehabilitation | no intervention-usual care | 2 | 254 | physical function | composite | SMD -0.16 (-0.66; 0.34) | no difference | 45 | Critically Low |
| Adamse 2018 | telerehabilitation | no intervention-usual care | 2 | 107 | physical function | composite | SMD 0.08 (-0.37; 0.53) | no difference | 27 | Critically Low |
| Adamse 2018 | telerehabilitation | no intervention-usual care | 3 | 507 | HrQoL | SF-36 overall | SMD 0.03 (-0.17; 0.23) | no difference | 23 | Critically Low |
| Adamse 2018* | telerehabilitation | no intervention-usual care | 3 | 425 | HrQoL | SF-36 (PCS) physical | MD 0.11 (-0.15; 0.37) | no difference | 42 | Critically Low |
| Adamse 2018* | telerehabilitation | no intervention-usual care | 3 | 426 | HrQoL | SF-36 (MCS) mental | MD 0.28 (-0.08; 0.64) | no difference | 70 | Critically Low |
| Cuenca-Martinez 2022a | telerehabilitation | no intervention-usual care | 38 | 5337 | pain | composite | SMD −0.17 (−0.26; −0.09) | favour intervention | 44 | Low |
| Cuenca-Martinez 2022a | telerehabilitation | no intervention-usual care | 13 | 1642 | pain | composite | SMD -0.24 (-0.44; -0.05) | favour intervention | 58 | Low |
| Cuenca-Martinez 2022a | telerehabilitation | In person | 5 | 486 | pain | composite | SMD 0.21 (0.15; 0.27) | favour control | 0 | Low |
| Cuenca-Martinez 2022a | telerehabilitation | no intervention-usual care | 3 | 340 | cognitive function | composite | SMD -0.57 (-1.08; -0-06) | favour intervention | 4 | Low |
| Cuenca-Martinez 2022a | telerehabilitation | no intervention-usual care | 16 | 1613 | cognitive function | composite | SMD -0.40 (-0.48; -0.32) | favour intervention | 31 | Low |
| Cuenca-Martinez 2022a | telerehabilitation | no intervention-usual care | 20 | 2811 | health literacy | composite | SMD 0.38 (0.23; 0.54) | favour control | 62 | Low |
| Cuenca-Martinez 2022b | telerehabilitation | no intervention-usual care | 32 | 3531 | emotional function | composite | SMD -0.35 (-0.46; -0.24) | favour intervention | 57 | Critically Low |
| Cuenca-Martinez 2022b | telerehabilitation | no intervention-usual care | 21 | 2578 | emotional function | composite | SMD -0.32 (-0.42; -0.21) | favour intervention | 37 | Critically Low |
| Cuenca-Martinez 2022b | telerehabilitation | no intervention-usual care | 4 | 789 | emotional function | composite | SMD -0.13 (-0.28; 0.02) | no difference | 0 | Critically Low |
| Dias 2021 | telerehabilitation | no intervention-usual care | 3 | 197 | pain | composite | SMD 0.1 (-0.2; 0.4) | no difference | 0 | Critically Low |
| Dias 2021 | telerehabilitation | no intervention-usual care | 4 | 223 | physical function | composite | SMD -0.1 (-0.6; 0.5) | no difference | 28,4 | Critically Low |
| Dias 2021 | telerehabilitation | no intervention-usual care | 5 | 575 | pain | composite | SMD -0.3 (-0.7; -0.1) | favour intervention | 46,2 | Critically Low |
| Dias 2021 | telerehabilitation | no intervention-usual care | 5 | 577 | physical function mixed PROMs+objective | composite | SMD 0.2 (-0.9; 1.3) | no difference | 65,7 | Critically Low |
| Dias 2021 | telerehabilitation | no intervention-usual care | 2 | 385 | HrQoL | composite | SMD 0.9 (-0.1; 1.8) | no difference | 0 | Critically Low |
| Chaudhry 2021 | mixed | In person | 6 | 581 | PREM treatment | patient satisfaction | OR 0.89 (0.40; 1.99) | no difference | 61 | Low |
| McDonnell 2022 | mixed | In person | 4 | 287 | PREM treatment | patient satisfaction | RR 0.98 (0.90; 1.07) | no difference | 0 | Critically Low |
| McDonnell 2022 | mixed | In person | 3 | 294 | physical function | WOMAC | SMD -0.12 (-0.35; 0.11) | favour intervention | 0 | Critically Low |
| McDonnell 2022 | mixed | In person | 6 | 397 | objective physical function | TUG | SMD -0.19 (-0.63; 0.25) | no difference | 73 | Critically Low |
| McDonnell 2022 | mixed | In person | 4 | 294 | pain | composite | SMD 0.33 (0.05; 0.61) | favour control | 25 | Critically Low |
| Oliveira 2018 | digital self-management | no intervention-usual care | 2 | 116 | pain | composite | SMD -0.50 (-1.91; 0.91) | no difference | 92 | Critically Low |
| Oliveira 2018 | digital self-management | no intervention-usual care | 2 | 116 | physical function | composite | SMD -0.81 (-2.34; 0.73) | no difference | 93 | Critically Low |
| **Osteoarthritis** | | | | | | | | | | |
| Agostini 2015 | telerehabilitation | no intervention-usual care | 3 | 248 | objective physical function | TUG | MD -5.17 (-9.79; -0.55) | favour intervention | 84 | Critically Low |
| Chen 2021 | telerehabilitation | no intervention-usual care | 10 | 1335 | pain | composite | SMD -0.29 (-0.48; -0.10) | favour intervention | 62 | Critically Low |
| Chen 2021 | telerehabilitation | no intervention-usual care | 11 | 1390 | physical function | composite | SMD 0.22 (-0.00; 0.43) | no difference | 73 | Critically Low |
| Chen 2021 | telerehabilitation | no intervention-usual care | 8 | 727 | HrQoL | composite | SMD 0.25 (0.04; 0.46) | favour intervention | 45 | Critically Low |
| Gazendam 2022 | telerehabilitation | In person | 3 | 282 | pain | VAS | MD -3.30 (-8.03; 1.43) | no difference | 84 | Critically Low |
| Gazendam 2022 | telerehabilitation | In person | 4 | 457 | physical function | composite | MD -2.87 (-6.03; 0.30) | no difference | 73 | Critically Low |
| Jiang 2018 | telerehabilitation | In person | 2 | 161 | pain | VAS | MD 0.52 (-0.20; 1,24) | no difference | 0 | Critically Low |
| Jiang 2018 | telerehabilitation | In person | 2 | 270 | physical function | WOMAC | MD -1.13 (-2.02; -0.23) | favour intervention | 0 | Critically Low |
| Jiang 2018 | telerehabilitation | In person | 2 | 198 | objective physical function | Degrees active flexion ROM | MD 2.40 (-0.34; 5.15) | no difference | 0 | Critically Low |
| Jiang 2018 | telerehabilitation | In person | 3 | 396 | objective physical function | Degrees active extension ROM | MD 0.30 (0.20; 0.40) | favour control | 0 | Critically Low |
| Jiang 2018 | telerehabilitation | In person | 2 | 198 | objective physical function | Quadriceps strenght | MD 2.82 (1.31; 4.32) | favour control | 0 | Critically Low |
| Schafer 2018 | telerehabilitation | no intervention-usual care | 6 | 742 | pain | composite | SMD -0.31 (-0.58; -0.04) | favour intervention | 67 | Critically Low |
| Schafer 2018 | telerehabilitation | no intervention-usual care | 4 | 479 | physical function | composite | SMD 0.30 (-0.17; 0.76) | no difference | 83 | Critically Low |
| Schafer 2018 | telerehabilitation | no intervention-usual care | 4 | 496 | HrQoL | composite | SMD 0.24 (0.05; 0.43) | favour intervention | 10 | Critically Low |
| Tsang 2022 | telerehabilitation | In person | 4 | 681 | pain | composite | SMD -0.15 (-0.47; 0.16) | no difference | 74 | Critically Low |
| Tsang 2022 | telerehabilitation | In person | 5 | 886 | objective physical function | Active ROM flexion | SMD -0.12 (-0.30; 0.07) | no difference | 45 | Critically Low |
| Tsang 2022 | telerehabilitation | In person | 4 | 699 | objective physical function | Active ROM extension | SMD 0.19 (0.04; 0.34) | favour control | 0 | Critically Low |
| Tsang 2022 | telerehabilitation | In person | 3 | 642 | physical function | composite | SMD -0.04 (-0.19; 0.12) | no difference | 0 | Critically Low |
| Wang 2021 | telerehabilitation | In person-usual care | 6 | 794 | pain | composite | SMD 0.11 (-0.10; 0.32) | no difference | 48 | Critically Low |
| Wang 2021 | telerehabilitation | In person-usual care | 5 | 547 | objective physical function | composite | SMD -0.54 (-1.08; -0.01) | favour intervention | 89 | Critically Low |
| Wang 2021 | telerehabilitation | In person-usual care | 3 | 558 | physical function | composite | SMD -0.01 (-0.18; 0.15) | no difference | 0 | Critically Low |
| Wang 2021 | telerehabilitation | In person-usual care | 5 | 652 | HrQoL | composite | SMD -0.09 (-0.26; 0.07) | no difference | 7 | Critically Low |
| Wang 2021 | telerehabilitation | In person-usual care | 3 | 512 | PREM treatment | composite | SMD -0.04 (-0.21; 0.14) | no difference | 0 | Critically Low |
| Wang 2021 | telerehabilitation | In person-usual care | 4 | 446 | objective physical function | ROM flexion | MD -0.65 (-2.48; 1.18) | no difference | 0 | Critically Low |
| Wang 2021 | telerehabilitation | In person-usual care | 4 | 446 | objective physical function | ROM extension | MD 0.38 (-0.40; 1.16) | no difference | 0 | Critically Low |
| Wang 2021 | telerehabilitation | In person-usual care | 3 | 512 | PREM treatment | patient satisfaction | SMD 0.04 (-0.14; 0.21) | no difference | 0 | Critically Low |
| Wang 2021 | telerehabilitation | In person-usual care | 3 | 389 | emotional function | mental heatlh | SMD -0.10 (-0.33; 0.13) | no difference | 11 | Critically Low |
| Wang 2019 | telerehabilitation | no intervention-usual care | 5 | 504 | pain | VAS | MD -0.25 (-0.48; -0.02) | favour intervention | 32 | Low |
| Wang 2019 | telerehabilitation | no intervention-usual care | 3 | 279 | objective physical function | TUG | MD -4.45 (-10.55; 1.64) | no difference | 96 | Low |
| Wang 2019 | telerehabilitation | no intervention-usual care | 3 | 560 | physical function | WOMAC subscale function | MD -0.09 (-0.22; 0.04) | no difference | 15 | Low |
| Wang 2019 | telerehabilitation | no intervention-usual care | 2 | 258 | objective physical function | 6MWT | MD -29.36 (-65.71; 6.99) | no difference | 88 | Low |
| Xie 2021 | telerehabilitation | In person | 4 | 411 | pain | WOMAC subscale | SMD -0.21 (-0.40; -0.01) | favour intervention | 0 | Critically Low |
| Xie 2021 | telerehabilitation | In person | 4 | 411 | physical function | WOMAC subscale | SMD -0.08 (-0.27; 0.12) | no difference | 0 | Critically Low |
| Safari 2020 | digital self-management | no intervention-usual care | 7 | 1614 | pain | composite | SMD -0.28 (-0.38; -0.18) | favour intervention | 0 | Moderate |
| Safari 2020 | digital self-management | In person | 3 | 762 | pain | composite | SMD -0.15 (-0.29; -0.01) | favour intervention | 0 | Moderate |
| Safari 2020 | digital self-management | no intervention-usual care | 7 | 1625 | physical function | composite | SMD -0.26 (-0.35; -0.16) | favour intervention | 0 | Moderate |
| Safari 2020 | digital self-management | In person | 3 | 764 | physical function | composite | SMD -0.04 (-0.18; 0.11) | no difference | 11 | Moderate |
| **Other disorders of the musculoskeletal system and connective tissue** | | | | | | | | | | |
| Esfandiari 2022 | telerehabilitation | no intervention-usual care | 6 | 272 | physical function mixed PROMs+objective | composite | SMD -0.33 (-0.75; 0.08) | no difference | 58 | Critically Low |
| Esfandiari 2022 | telerehabilitation | no intervention-usual care | 2 | 167 | HrQoL | composite | SMD 0.08 (-0.15: 0.30) | no difference | 82 | Critically Low |
| Esfandiari 2022 | telerehabilitation | no intervention-usual care | 5 | 149 | social function | antecedents of behavior | SMD -0.04 (-0.36; 0.28) | no difference | 0 | Critically Low |
| Gava 2022 | telerehabilitation | In person | 2 | 52 | pain | composite | SMD 0.35 (-0.82; 1.52) | no difference | NA | Low |
| Gava 2022 | telerehabilitation | In person | 2 | 52 | physical function | composite | SMD 0.60 (-0.32; 1.53) | no difference | NA | Low |
| Gava 2022 | telerehabilitation | no intervention-usual care | 2 | 172 | physical function | composite | SMD 0.43 (-0.97; 0.11) | no difference | NA | Low |
| Lin 2021 | telerehabilitation | In person | 4 | 117 | objective physical function | composite | SMD -1.54 (-2.86; -0.21) | favour control | 89 | Critically Low |
| **Other dorsopathies** | | | | | | | | | | |
| Lara-Palomo 2022 | telerehabilitation | In person | 3 | 147 | pain | 0-10 scale | MD -0.59 (-1.77; 0.59) | no difference | 81 | Critically Low |
| Lara-Palomo 2022 | telerehabilitation | In person | 3 | 97 | physical function | composite | SMD -0.20 (-0.81; 0.41) | no difference | 45 | Critically Low |
| Lara-Palomo 2022 | telerehabilitation | In person | 2 | 50 | HrQoL | composite | SMD ranged from -0.70 to 0.43, 95% CI ranged from -2.23 to 1.96 | no difference | 0 to 83 | Critically Low |
| Lara-Palomo 2022 | telerehabilitation | no intervention-usual care | 2 | 575 | pain | composite | SMD -0.64 (-1.72; 0.45) | no difference | 97 | Critically Low |
| Lara-Palomo 2022 | telerehabilitation | no intervention-usual care | 2 | 956 | physical function | composite | SMD -0.39 (-0.87; 0.09) | no difference | 87 | Critically Low |
| Du 2020 | digital self-management | no intervention-usual care | 5 | 753 | pain | composite | SMD -0.16 (-0.30; -0.02) | favour intervention | 0 | Critically Low |
| Du 2020 | digital self-management | no intervention-usual care | 5 | 753 | physical function | composite | SMD -0.25 (-0.40; -0.11) | favour intervention | 28 | Critically Low |

Legend

6MWT, 6 Minute Walking Test; AMSTAR, Measurement Tool to Assess Systematic Reviews; CI, Confidence Interval; HRQoL, Health Related Quality of Life; MCS, mental component summary; MD, Mean Difference; PCS, physical component summary; PREMs, Patient-Reported Experience Measures; PROMs, Patient-Reported Outcome Measures; ROM, Range Of Motion, SF-36, Short Form Health Survey 36; SMD, Standardized Mean Difference; TUG, Time Up and Go; VAS, Visual Analogue Scale; WOMAC, Western Ontario and McMaster Universities Osteoarthritis Index

*subgroup scales of SF-36
