# Supplementary material for: Effectiveness of Telemedicine for Musculoskeletal Disorders: Umbrella Review
Source: J Med Internet Res. 2024 Feb 2;26:e50090. doi: 10.2196/50090 (PMC10873802; doi:10.2196/50090)
Supplement: Multimedia Appendix 3 [file jmir_v26i1e50090_app3.docx]

## **Multimedia Appendix 3. Narrative synthesis of patient-reported outcomes and experiences**

**1 PROMs**

- 1. **Pain**

No differences in pain intensity were reported by Chaudry et al. 2021 from patients receiving telemedicine or conventional in-person care, measured by Visual Analog Scale (VAS) and Revised Faces Pain Scale.

Fritsch et al. 2020 investigated trials comparing combined telerehabilitation and telemedicine interventions with usual care, finding different patient-reported outcomes, measured with the Western Ontario and McMaster Osteoarthritis Index (WOMAC) - Pain domain, Numeric Rating Scale (NRS) and VAS. Compared with usual care alone, adding a text message did not significantly improve pain in patients with frozen shoulder, nor pain at rest or during walking after knee arthroplasty. Text message reminders added to a smartphone-based rehabilitation, after a multidisciplinary inpatient program, did not improve chronic widespread pain, compared with the same intervention without text messages. However, a text message to increase light physical activity in addition to motivational counseling led to significant, beneficial effects on pain compared with usual care in patients affected by rheumatoid arthritis. Lastly, text message reminders added to health coaching alone decreased the frequency of knee pain exacerbations in female patients.

Hewitt et al. 2020 investigated trials comparing digital health interventions with different control groups, including no intervention (waiting list), standard means of delivery (i.e., face-to-face, class-based, printed materials, hand outs), non-digital self-management interventions (i.e., leaflets) and non-interactive digital interventions (i.e., web page or flat copy). Pain outcomes were expressed by WOMAC - Pain domain, NRS, VAS, EuroQuol-5 Dimension (EQ-5D) - Pain domain, Knee Injury and Osteoarthritis Outcome Score (KOOS) – Pain domain, Modified Von Korff scale – Pain domain and a yes/no back pain binary question. Nine studies reported significant improvements, five of which by participants with LBP, one with osteoarthritis, one with chronic hip and knee pain, one with knee pain. In two of these studies, improvement was not maintaned at the last time point measured. Ten studies included by the review did not demonstrate better outcomes reported by patients undergoing telerehabilitation in comparison to control groups, one of which by participants with knee osteoarthritis, six with chronic LBP, two with chronic musculoskeletal pain and one with work-related neck and upper limb disorders.

All the studies included by Hussain et al. 2022 reported positive evidence in that digital technologies improve the patients’ ability to manage chronic pain, compared with non-specified control groups and expressed by NRS, VAS, Pain Responses Self-Statements (PRSS) and Wisconsin Brief Pain Questionnaire.

Jansson et al. 2020a compared telerehabilitation with conventional rehabilitation (in-person outpatient physical therapy), reporting no significant difference in VAS pain intensity by patients who underwent lower limb joint replacement. A beneficial, significant effect was reported about the same intervention, by the same population, when expressed by NRS at rest, NRS in motion and narcotic drugs consumption.

Jansson et al. 2020b investigated pain by means of telephone follow-up survey and VAS, in patients with frozen shoulder, shoulder instability surgery or patients who underwent low- and medium-intensity orthopedic surgery. Compared with conventional care, telephone-delivered interventions showed no significant difference in pain intensity.

Latif-Zade et al. 2021 found no statistically significant differences between telerehabilitation and office-based rehabilitation in pain intensity reported by patients affected by knee osteoarthritis. Both groups improved their scores in WOMAC – Pain dimension from baseline to end of the study.

No pain variations were also reported by one moderate and two poor quality studies included in the synthesis of Master et al. 2022, expressed by EQ-5D – Pain dimension, KOOS – Pain dimension and Mc Gill Pain Questionnaire, comparing telerehabilitation with different control groups (in-person, usual care, home exercise, use of wearables without feedback).

Three studies that compared telerehabilitation with active controls suggested that remote exercise programs were not inferior to in-person physical therapy in patients affected by knee osteoarthritis, building on the systematic review conducted by McHugh et al. 2022. Six studies in the same synthesis compared telerehabilitation with passive comparators, reporting mixed results. Pain intensity was assessed with WOMAC – Pain dimension, NRS, VAS and KOOS – Pain dimension.

Pain intensity was reported in three of the six studies included by the investigation of Nicholl et al. 2017 on patients with LBP. Only one study reported beneficial effects in favor of a digital self-management program over usual care, over digital non-interactive or over non-digital, self-management control groups, sixteen weeks after intervention. Pain intensity was expressed by NRS and VAS, though part of a composite measure including duration and frequency.

Safari et al. 2020 compared the effects of digital-based, self-management programs with those of usual care controls on pain outcomes, reported by patients affected by osteoarthritis. Seven studies reported medium effect, significant benefits in favor of the intervention; three studies reported long-term, small but significant benefits in favor of the intervention; two studies demonstrated no significant variation. Pain outcomes were assessed by using the Arthritis Impact Measurement Scales 2 (AIMS 2) – Pain domain, WOMAC – Pain domain, HOOS – Pain domain, KOOS – Pain domain and NRS. The only study comparing the experimental intervention with a health education, control group also reported medium effect, significant benefits in favor of the former. In this case, pain outcomes were assessed by using the AIMS 2 – Pain domain.

One trial included by Srikesavan et al. 2018 compared a web-based, self-management care program with a waiting list control, showing a small, non-significant beneficial effect in favor of the former, at short- and medium-term, measured with the AIMS 2- Pain domain. Another trial included by the same review compared the same intervention with a usual care control group, showing a small, non-significant variation from baseline at long-term in favor of the intervention, measured with a Visual Numeric Scale.

Mobile health solutions for LBP were compared with physical therapy and usual care by seven studies reported by Tabacof et al. 2021, employing a yes/no binary questionnaire on neck pain, a yes/no binary questionnaire on upper back pain and the Graded Chronic Pain scale. Six studies found the intervention group to achieve a significant reduction in self-reported pain.

**1.2 Health-related quality of life**

Generic Function

No differences in generic function were reported by Chaudry et al. 2021 between telemedicine (any form of remote or virtual care including video, telephone, or internet-based care intervention) and in-person visits control group, in a mixed population undergoing orthopedic care, both measured with Short-Form-12 (SF-12) and EQ-5D questionnaires.

General Health

No differences in general health were reported by Master et al. 2022 between telerehabilitation with wearables and control groups, in a mixed population of adults following orthopedic surgical treatment, both measured with Short-Form 36 (SF-36) and EQ-5D questionnaires.

Quality of Life

No statistically significant differences were reported by Cuevas-Lara et al. 2021 between game-based, telerehabilitation interventions and conventional rehabilitation control groups, in a population of hospitalized elderly patients, measured with the EQ-5D questionnaire.

Fritsch et al. 2020 compared combined telerehabilitation and telemedicine interventions with usual care, in patients suffering from musculoskeletal pain, measuring quality of life with the Mental Component Summary Score (MCS) and the Physical Component Summary Score (PCS) of Short-Form 8 (SF-8) and SF-36 questionnaires. In patients affected by chronic widespread pain, adding text messages to smartphone-based rehabilitation after a multidisciplinary inpatient rehabilitation program did not increase the physical component of quality of life, but improved the mental component, when compared with inpatient rehabilitation alone. In patients affected by rheumatoid arthritis, adding text message reminders to motivational counseling sessions resulted in greater improvement in both physical and mental components, when compared with usual care. No other differences were found.

Five studies reported by Hewitt et al. 2020 described statistically significant improvements in the quality of life of patients affected by chronic, acute and subacute musculoskeletal pain, measured with the Assessment Of Quality of Life 2 (AQOL2), EQ-5D and Quality Of Life Inventory (QOLI) questionnaires.

All the studies analysed by Hussain et al. 202 reported statistically significant benefits of digital health technologies in the management of chronic pain, regardless to different control groups, measured with AQOL2, SF-36 and Pediatric Quality Of Life Inventory Questionnaires.

Significant improvements in quality of life were reported by a study included in Jansson et al. 2020a, which compared internet-based, integrative rehabilitation on a home care platform and traditional nursing care control group, in patients discharged after total knee and hip replacement, measured with the SF-36 questionnaire, 4 months after intervention, but not after 6 or 12 months.

Significant improvements in quality of life were also reported by a study included in Jansson et al. 2020b, which compared additional telephone-delivered nursing support to conventional physical therapy, in a population who underwent shoulder instability surgery, measured with the Quick Disability of the Arm, Shoulder and Hand (QuickDASH) Score. However, the difference between groups at 4 months disappeared at 6 and 12 months.

Master et al. 2022 found one moderate and two poor quality studies to compare telerehabilitation with control groups (activity tracker with/without feedback, exercise program with/without fitness trackers, telemonitored walking or not), in adults undergoing orthopedic surgery, measured with KOOS – Quality of life domain, SF-36 and EQ-5D, showing no difference in quality of life.

A composite score adopted in one study included by Nicholl et al. 2017 reported beneficial interventions from a digital, interactive self-management tool in the quality of life of adult patients affected by LBP. The score was adapted from the Multidimensional Pain Inventory Interference Scale, the Interference Scale of the Brief Pain Inventory and the Dartmouth Primary Care Cooperative Project (COOP).

Oliveira et al. 2018 investigated the effect of physical activity interventions using electronic feedback (i.e., physical activity monitors) on pain and disability, compared to minimal or no interventions, in patients affected by chronic musculoskeletal pain. Quality of life was measured both in general terms and in specific domains by using SF-36 and EQ-5D questionnaires. In general terms, one study found greater improvement in favor of the minimal intervention group at short-term follow-up but no differences at intermediate follow-up. In terms of bodily pain, vitality, physical functioning, role emotional and mental health, another study found significant benefits at intermediate follow-up in favor of the digital intervention, compared to minimal intervention.

No significant improvements were reported by Safari et al. 2020 between an internet-based self-management program compared with no intervention (waiting list) in patients affected by osteoarthritis, in terms of quality of life, neither 4 months nor 12 after the digital self-management intervention, measured with quality of life domains of the KOOS and Hip Injury and Osteoarthritis Outcome Score (HOOS).

Srikesavan et al. 2018 investigated the effect of web-based telerehabilitation in comparison to different control groups, including no intervention (waiting list for surgery), on patients affected by rheumatoid arthritis. Quality of life was measured with Rheumatoid Arthritis Quality of Life (RAQoL) questionnaire, Reseach AND development (RAND)-36 questionnaire, Quality Of Life Scale (QOLS) and AIMS 2. One study compared a web-based, individualised physical activity training with web-based, general information on physical acitivty only, finding small, non-significant effects in the short-term but significant changes in the medium- and long-term. Another study compared the benefits of a web-based self-management program with waiting lists, finding small, non-significant effects at both short- and medium- term.

Wang et al. 2019 investigated the effect of telerehabilitation, game-based or web-based therapy in comparison to usual care control groups, measuring quality of life with the SF-36 questionnaire. Two trials on patients who underwent telerehabilitation after total knee replacement (TKR) showed signifcant improvements in mental component and physical function subscales. One study on patients who underwent total hip replacement (THR) found all the physical function, general health and mental health subscales of the SF-36 to be significantly higher in the telerehabilitation group after 3 months, but non-significant at 9 months follow-up.

**1.3 Health Literacy**

Interest to surgery

Pain management digital care programs before intervention can reduce the interest of patients affected by chronic musculoskeletal pain towards surgery, Hewitt et al. 2020 reports. Interest to surgery was evaluated in two RCTs by means of a scale from 0 to 10.

Self-efficacy

Two studies reported statistically significant improvements in the sef-efficacy of patients undergoing telerehabilitation support for musculoskeletal pain, measured with the Arthritis Self-Efficacy Scale (ASES), Hewitt et al. 2020 reports. The intervention groups were an online self-help cognitive behavioral therapy program and an internet-delivered exercise program.

All the studies included by Hussain et al. 2022 also reported statistically significant benefits in favor of the digital management of chronic pain, measured with ASES, Pain Self-Efficacy Questionnaire (PSEQ) and Children Arthritis Self-Efficacy (CASE) Score.

Nicholl et al. 2017 included trials comparing interactive digital interventions with different control groups, measuring self-efficacy outcomes with different questionnaires. All patients were affected by LBP. A favorable effect for the digital intervention group in comparison to the waiting list control was found through the Self-Efficacy for Exercise Scale (SEES). A self-developed self-efficacy scale included in a composite outcome measure also reported a beneficial difference in favor of the digital intervention, compared with minimal digital intervention and usual care. Two other studies comparing web-based complementary intervention to standard care or pedometer only found no differences in self-efficacy measured by PSEQ and Exercise Regularly Scale (ERS).

Srikesavan et al. 2018 also compared the self-efficacy of a web-based self-management program against waiting list or usual care, measured with the ASES, in patients affected by rheumatoid arthritis. Better effects in favour the former were found in the short- and medium-term when compared with the waiting list, while small but significant, better long-term effects were found in favour of the former when compared with usual care.

Self-care

Nicholl et al. 2017 included trials comparing telerehabilitation interventions with different control groups, measuring self-care with 14 different outcomes, most of which (10) were reported in only three studies, two of which are probably overlapping. All the population were patients affected by LBP. According to the authors, the digital intervention reported bettere benefits on 8 different outcomes.

Coping ability

Coping ability improved significantly when telerehabilitation interventions were compared to usual care controls, non-interactive digital controls or waiting lists, Hewitt et al. 2020 reports. The outcomes were drawn from six studies on patients affected by chronic pain, and measured with the Coping Strategies Questionnaire (CSQ).

All the studies analysed by Hussain et al. 2022 reported statistically significant benefits in favor of digital health technologies in the management of chronic pain, and one of the main benefits was the enhancement of pain coping skills. Coping skills were measured with CSQ, Pain Coping Questionnaire and a single domain from the Hert Hope Index.

No differences in coping strategies were reported by Jansson et al. 2020b between a telephone-delivered intervention and conventional care, in the treatment of patients with chornic LBP, measured with the CSQ.

One study reported by Nicholl et al. 2017 described between-group differences in support of an interactive self-management website against standard text-based materials, in patients affected by LBP, measured by the Chronic Pain Coping Inventory.

**1.4 Cognitive Function**

Pain Catastrophizing

Four studies analyzed by Hewitt et al. 2020 reported pain catastrophizing to improve significantly in patients affected by chronic pain treated with a telerehabilitation intervention. The intervention was compared against multiple control groups and measured with Pain Catastrophizing Scale (PCS), pain catastrophizing subscales of CSQ and Graded Chronic Pain Scale.

All the studies analysed by Hussain et al. 2022 reported statistically significant benefits of digital health technologies in chronic pain management. Multiple comparison groups were considered against telerehabilitation interventions and measured with PCS, Pain Responses Self-Statement and Chronic Pain Acceptance 8 questionnaire.

One study reported by Nicholl et al. 2017 described a beneficial effect of a digital intervention group in comparison with a waiting list control in the treatment of patients affected by LBP, measured with all PCS subscales (rumination, magnification and helplessness).

Kinesiphobia

All the studies analysed by Hussain et al. 2022 reported statistically significant benefits of digital health technologies in chronic pain management. Multiple comparison groups were considered against telerehabilitation interventions and measured with the Tampa Scale of Kinesiphobia (TSK).

Impression of change

One study reported by Nicholl et al. 2017 described between-group differences favoring an interactive self-management website against standard text-based materials, in patients affected by LBP, measured by the Patient Global Impression of Change.

Fear avoidance

Nicholl et al. 2017 included trials comparing telerehabilitation interventions with different control groups all dedicated to patients affected by LBP. One study reported an effect in favor of online cognitive behavioral therapy, compared to waiting list, measured with the physical function subscale of the Fear Avoidance Belief Questionnaire. One study did not report any difference between digital intervention, minimal digital intervention and usual care measured with the TSK.

Disease Knowledge

Nicholl et al. 2017 compared LBP treatments also in terms of disease knowledge from patients. Two studies did not find any difference between digital intervention and control group, both using a self-developed questionnaire, both comparing an individually-tailored informational support with general instruments of patient information. Another study employed a self-developed composite score in which digital intervention, minimal digital intervention and usual care were compared, finding favorable outcomes in support of the digital intervention group.

One study reported by Srikesavan et al. 2019 found a significant, beneficial effect in patients rheumatoid arthritis knowledge comparing different experimental online supports with gamification against a control group with no access to the website, favoring the intervention group 2 and 4 months after the intervention had begun. The outcome was measured with rheumatoid arthritis Patient Knowledge Questionnaire (PKQ).

**1.5 Physical Function**

Patient-Reported Function

No differences in any measure of disease-specific function, measured with WOMAC and Morrey Outcome Scale were reported by Chaudry et al. 2021 comparing remote interventions and in-person orthopedic consultations.

In patients affected by chronic widespread pain, Fritsch et al. 2020 found that adding text messages to smartphone-based rehabilitation after a multidisciplinary inpatient rehabilitation program did not improve function in patients affected by frozen shoulder disorder, measured with Patient Specific Functional Scale, Activities of Daily Living (ADL) Scale and Health Assessment Questionnaire.

Hewitt et al. 2020 found 19 RCTs comparing physical function between telerehabilitation interventions and different control groups dedicated to chronic pain patients. The outcomes employed were WOMAC, 7-Items Likert scale, HOOS, KOOS and SF-36 subscales, Roland Morris Disability Questionnaire, Modified Oswestry Disability Index (ODI), Oswestry Disability Questionnaire, Multidimensional Pain Inventory Interferences Scale, Interference Scale of the Brief Pain Inventory, Dartmouth COOP, Hannover Functional Ability Questionnaire, Fibromyalgia Impact Questionnaire. 10 studies reported statistically significant improvements in favor of the intervention group, among which 5 were not maintained at the last point of measure. 6 studies reported effect sizes (3 large, 2 moderate, 1 small).

All the studies analysed by Hussain et al. 2022 reported statistically significant benefits of digital health technologies in chronic pain management. Multiple comparison groups were considered against telerehabilitation interventions and physical function was measured with PSFS, World Health Organization Disability Assessment Scale (WODAS), WOMAC, KOOS, Simple Shouder Test (SST), Physical Activity Scale Questionnaire, Roland Morris Disability Questionnaire and AIMS 2 subscale.

The results found by Jansson et al. 2020a on patients who underwent TKA and THA were more heterogeneous. Telerehabilitation showed significant improvements on PSFS, Harris Hip Score and Knee Society Score in comparison with conventional in-person outpatient physical therapy, but no significant difference in KOOS, in one study. Conflicting results were found on WOMAC. Overall, the effect of rehabilitation on stiffness, function and total score was positive but moderate.

Jansson et al. 2020b compared mixed telerehabilitation and telemedicine interventions with conventional rehabilitation programs in patients affected by different orthopedic disorders. Shoulder function was evaluated in patients with frozen shoulder, subacromial impingement and shoulder instability surgery; compared with conventional care, computer-delivered intervention showed no difference in SST and Rowe Score; compared with conventional care, telephone-delivered interventions showed significant benefits in Oxford Shoulder Instability Score. One study evaluated the effects of interventions on disability in patients affected by LBP, using the Roland Morris Disability Questionnaire: compared with conventional care, a telephone-delivered intervention showed significant difference with a medium-to-strong effect size.

Two studies included by Jiang et al. 2016 reported significant improvements in WOMAC outcomes to the benefit of patients undergoing telerehabilitation after TKA. Face-to-face conventional visits (both at home and outpatient) were compared to remote rehabilitation at home.

Latif-Zade et al. 2021 reported evidence for the same efficacy of telerehabilitation compared to office-based rehabilitation in terms of WOMAC improvement in patients affected by knee osteoarthritis.

Master et al. 2022 found that physical activity interventions based on wearables did not add any benefit in terms of physical function, compared with conventional care control groups, 6 months after orthopedic surgery, measured with KOOS, WOMAC and ODI.

Petersen et al. 2019 investigated the benefits of mixed telerehabilitation and telemedicine in orthopedics. All the studies included in the synthesis reported WOMAC improvements without differences at final follow-up. One study did not report any statistical difference between intervention (telemedicine support to further accelerate discharge in fast-track THR) and control group (ordinary fast-track THR) at 3 months follow-up, measuring physical function with the Oxford Hip Score.

One study included by Srikesavan et al. 2018 showed no significant benefits in patients affected by rheumatoid arthritis undergoing web-based physical activity training or receiving web-based general information, measuring physical function with the Health Assessment Questionnaire. Another study included in the same review compared web-based self-management to usual care, also reporting no significant differences in function between groups, at long-term, measured with the McMaster Toronto Arthritis Patient Preference Questionnaire.

Wang et al. 2019 reported low-to-moderate quality in the assessment of physical function measured with WOMAC in patients undergoing telereheabilitation after THR and TKR, but found significant improvements after TKA in patients undergoing nurse-based, telephone follow-up after discharge, measuring physical function with the relative SF-36 subscale, in.

Activities of Daily-Living

Internet-based home rehabilitation showed significant improvements in ADL measured with the Barthel Index, compared with routine nursing care, in one study on patients who underwent hip replacement surgery, Jansson et al. 2020a reports.

ADL were also evaluated in patients with chronic LBP using the Patient Reported Outcome Measurement Information System (PROMIS) Health Assessment Questionnaire. Compared with conventional care, telephone-delivered intervention showed significant benefits with a medium-to-strong effect size, Jansson et al. 2020b reports.

Disability

According to Nicholl et al. 2017 the literature is too heterogeneous to understand which digital self-management, digital non-interactive self-management, or non-digital self-management interventions work best for patients affected by chronic LBP, in what circumstances, in terms of pain-related physical disability, measured with ODI and Roland Morris Disability Questionnaire.

A minimal effect size in favor of an internet-based self-management program compared with usual care was reported by one study included in Safari et al. 2020, on patients affected by arthritis or fibromyalgia, measured with the Disability Health Assessment Questionnaire. However, the effect of the intervention did not persiste after 12 months follow-up.

**1.6 Emotional Function**

Anxiety

One study included by Hussain et al. 2020 demonstrates significant improvements after clinician-guided internet-delivered cognitive behavior internet therapy in reducing the anxiety associated with chronic pain, in comparison to a waiting list control group, measured with the General Anxiety Disorder 7-item questionnaire. Another study included in the same review reports small beneficial effects in terms of reduced anxiety associated with osteoarthritis pain, measured with the Pain Anxiety Symptoms Scale.

Psychological distress

Master et al. 2022 found that physical activity interventions based on wearables did not add any benefit in terms of psychological distress, compared with conventional care control groups, six months after orthopedic surgery, measured with the SF-36.

Depression

Nicholl et al. 2017 investigated the benefits of digital-self management interventions on patients affected by LBP. In terms of depression, one study reported the beneficial effects of a pilot online cognitive and behavioral therapy program against a waiting list control group, measured with the Negative Mood Regulation Scale.

Oliveira et al. 2018 investigated the benefits of digital self-management interventions based on electronic feedback, on patients suffering from chronic musculoskeletal pain. In terms of depression, one study compared a telesupervised aerobic exercise program for women affected by fibromyalgia with usuale care, finding non-significant benefits in favor of the intervention at intermediate follow-up, measured with a Visual Analog Scale.

**1.7 Social Function**

Behavior change

One study included by Cuevas-Lara et al. 2021 reported positive behavioral change in hospitalized elderly patients undergoing serious games-based rehabilitation to increase physical activity. The patients were administered a questionnaire in which they reported to feel more active, confident and painless in comparison to standard rehabilitation control group.

Work-related outcomes

One study included by Nicholl et al. 2017 evaluated return to work through a composite scale drawn from Stanford Presenteeism Scale, Work Limitations Questionnaire and actual time off work, after patients were given a mobile web-app to self-manage LBP, reporting beneficial effects in the intervention group.

Another study included by Petersen et al. 2021 evaluated return to work after THR and TKR, in patients undergoing 3-months integrative telerehabilitation or conventional physical therapy, finding self-reported positive results in favor of the experimental intervention.

**1.8 Adherence**

Adherence to treatment

Significant differences were found between game-based telerehabilitation and conventional rehabilitation groups by Cuevas-Lara et al. 2021, in terms of adherence to treatment, reporting higher values in the control group, measured by the duration of training activities directly recorded by patients. Evidence in favor of conventional rehabilitation was drawn from a single RCT in geriatric settings.

Adherence to chronic LBP treatment rates varied significantly across the studies included by Du et al. 2020, who underlines how to attract users is crucial to increase the impact of e-Health design. Time saving, interestingness and information sharing are strongly recommended by the reviewers to achieve more patient engagement.

One study included by Joansson 2020b showed a telephone-delivered intervention to increase compliance towards shoulder exercise in comparison to conventional care, in patients affected by frozen shoulder disorder, measured with the Shoulder Exercise Self-Reported Form.

No statistically significant effect of a web-based, patient dialogue intervention could be found on either involvement in decision-making, decision regret or treatment adherence 3 months after baseline, in patients affected by acute LBP, expressed by a single treatment questionnaire, according to a study included in Nicholl et al. 2017.

The poor return of exercise diaries prevented one study included by Esfandiari et al. 2022 to analyze adherence in patients who underwent lower limb amputations. Another study included in the same review showed favourable adherence rates to conventional rehabilitation in comparison to telerehabilitation for patients affected by phantom limb chronic pain, as a consequence of technical problems, insufficient instructions given by therapists on how to use the platform and pain being already reduced by early traditional treatment. Adherence was measured with self-reported activity on registers and logs.

Positive effects on adherence were recorded by Fritsch et al. 2020 when text messages were added to usual care in patients suffering from frozen shoulder and rheumatoid arthritis, using a Visual Analog Scale and the Compliance Questionnare for Rheumatology to express this outcome.

The majority of studies analysed by Hussain et al. 2022 reported increased adherence to exercise as another essential advantage of telerehabilitation, in the management of chronic pain, expressed with the Exercise Adherence Rating Scale.

One study included by Wang et al. 2019 showed the average time of daily home exercise in the telerehabilitation group (structured telephone follow-up) to be significantly higher than in the control, in patients affected by frozen shoulder disorder through a self-developed two-items reporting form. Two studies included in the same review showed no differences in the number of exercise sessions concluded daily, the former comparing home telerehabilitation with face-to-face home care after TKA, the latter comparing internet-based rehabilitation with conventional outpatient physical therapy after TKA, self-reported by patients on a diary.

**1.9 Side Effects**

Adverse events and complications

The majority of secondary outcomes evaluated by one study included by Esfandiari et al. 2022 reported the lack of significant differences between telerehabilitation, simpler telehealth and usual care, including adverse events rates self-reported by patients on activity registers.

Only one study evaluated adverse events in the review of Jansson et al. 2020a, equally reporting the absence of significant differences even when adverse events were controlled by health care professionals. In this case, telerehabilitation was compared with face-to-face home visits.

Three studies included in Jansson et al. 2020b confirmed the absence of self-reported complications and adverse events variations in patients undergoing orthopedic surgery or suffering from LBP, either in the computer- and telephone-delivered interventions or in the traditional control group.

Tsang et al. 2022 found similar hospital readmissions for knee-related problems both in telerehabilitation and conventional control groups, sometimes reported by patients, sometimes confirmed by medical records. Wang confirmed the same findings in patients who underwent THR and TKR.

No difference in the duration of consultation and complications rate were finally found by Petersen et al. 2021 when comparing orthopedic patients who underwent mixed telerehabilitation and telemedicine with conventional control groups.

Jiang et al 2016, Master et al. 2022 and Srikesavan et al. 2018 did not find information on adverse events, although it was among the subjects of systematic investigation.

**2 PREMs**

**2.1 Technology**

Usability

Three studies included in Adamse et al. 2018 analyzed usability and feasibility of different telerehabilitation interventions: one individually tailored exercise programme by computer with webcam recordings and teleconference feedback, one smartphone intervention, one web-based intervention. All the interventions were meant to treat chronic musculoskeletal pain and found the participants satisfied, valuing interactive features and support as most important. Patient experiences were investigated with Systems Usability Scale, a single question on usefulness and Client Satisfaction Questionnaire.

Enjoyment

One study included by Cuevas-Lara et al. 2021 compared the benefits of geriatric care exergames against self-regulated exercise, including enjoyment, measured with a Five-levels Likert Scale. Enjoyment was superior in the intervention group though non-statistically significant.

User experience

Wang et al. 2019 found a positive trend towards telerehabilitation after hip and knee replacement, in terms of improved adherence to physical activities and compliance to rehabilitation programs. Four trials reported user experience and showed similar levels of satisfaction between telerehabilitation interventions and usual care controls. More in detail, one trial found positive user experiences such as clear instructions, ease of taking or sharing a video and ease of seeing their progress about the asynchronous video-based software platform. Positive feedback from participants and therapists was reported also on a computer-aided multimedia training software in orthopedic rehabilitation. The outcomes were self-reported (i.e., on diaries).

**2.2 Treatment**

Patient Satisfaction

All the studies analysed by Hussain et al. 2022 reported statistically significant benefits of digital management of chronic pain, including patient satisfaction, measured with Hospital Consumer Assessment of Healthcare Providers and Systems Survey and Client Satisfaction Questionnaire Revised.

Motivation

One study included by Cuevas-Lara et al. 2021 compared the benefits of geriatric care exergames against self-regulated exercise, including motivation, measured with a Five-levels Likert Scale. Motivation was superior in the intervention group.

**3. Cost**

Seven SRs assessed this outcome, showing that telemedicine cost significantly less than in person visits/usual care. Details are reported in Table S1.

**Table S1.**

| SR review | N primary studies | Findings |
| --- | --- | --- |
| Gazendam 2022 | 1 | VR-based rehabilitation  costs significantly less than traditional rehabilitation median in United States dollars: 1050 vs. 2805, *p* < 0.001. |
| Jansson 2020a | 4 | The inperson outpatient physical therapy, telerehabilitation showed a significant decrease in the total cost (MD: US$_263; 95% CI: US$_382–US$143;p<0.001; significant decrease in the cost per treatment(MD: US$_12.09; 95% CI: US$_20.90–US$_3.20; p ¼ 0.08). The difference in the costs, however, was  only significant when the distance from home to the healthcare center was more than 30 km (US$81.3 (SD  ¼13.19) vs. US$102.7 (SD ¼19.5), p¼0.02). |
| Nicholl 2017 | 0 | No studies reported on health care costs or cost-effectiveness. |
| Petersen 2021 | 2 | Cost effectiveness of telemedicine applications for videoconsulations depending on the workload.   - In the Norwegian study, video consultation was more cost effective after 151 patient consultations per year - In the Finish study, the cost of the video consultation was lower after a workload of more than 80 patients per year. |
| Safari 2020 | 2 | - Per-participant costs were US $107 (range US $100-US $121) for osteoarthritis self-management and US $51 (range US $47-US $60) for health education. This cost was not compared with that of usual care. - No significant differences in healthcare utilization. |
| Tsang 2022 | 2 | Overall utilization of hospital-based resources was 60% less in the telerehabilitation group than for the  traditional face-to-face group. The median total costs in the telerehabilitation group were significantly lower than  in the control group (median $1050 vs. $2805, P < 0.001). |
| Jansson 2020b | 1 | Computer delivered interventions showed statistical significant differences in direct and indirect cost compared with conventional care. |
